# Gut Mycobiota Dysbiosis in Pulmonary Tuberculosis Patients Undergoing Anti-Tuberculosis Treatment

**DOI:** 10.1128/spectrum.00615-21

**Published:** 2021-12-15

**Authors:** Demin Cao, Weihua Liu, Na Lyu, Boxing Li, Weibo Song, Yanxiao Yang, Jianliang Zhu, Zhiguo Zhang, Baoli Zhu

**Affiliations:** a CAS Key Laboratory of Pathogenic Microbiology and Immunology, Institute of Microbiology, Chinese Academy of Sciences, Beijing, China; b University of Chinese Academy of Sciences, Beijing, China; c Shijiazhuang Center for Disease Control and Prevention, Hebei, China; d Gaocheng District Center for Disease Control and Prevention in Shijiazhuang, Hebei, China; e Gaocheng People's Hospital in Shijiazhuang, Hebei, China; f Beijing Changping Institute for Tuberculosis Prevention and Treatment, Beijing, China; g Department of Pathogenic Biology, School of Basic Medical Sciences, Southwest Medical University, Luzhou, China; Nanchang University

**Keywords:** gut mycobiota, anti-tuberculosis treatment, pulmonary tuberculosis

## Abstract

Patients with pulmonary tuberculosis (TB) undergoing anti-tuberculosis (anti-TB) treatment were previously reported to present gut bacterial microbiota dysbiosis, but the role of the mycobiota has not been reported. Here, we conducted a follow-up study of 29 naive TB patients who received first-line anti-TB drug treatment; we collected their fecal samples at different time points, as well as 22 fecal samples from healthy subjects. Fungal ITS2 and bacterial 16S rRNA amplicon sequencing were used to analyze the effects of active TB and anti-TB treatment on the gut microbiota. We found that naive TB patients had bacterial and fungal dysbiosis with altered community composition and a decreased density of the transkingdom correlation network. Anti-TB drug treatment significantly decreased the diversity of bacteria and fungi with altered composition. Notably, we observed that the abundance of Purpureocillium lilacinum tended to decrease and *Nakaseomyces* spp. tended to increase in the anti-TB treatment, and all of them had increased proportions in the three TB groups compared with healthy subjects. We found that the fungal-bacterial transkingdom network was severely altered in TB patients after 2 months of treatment, and new fungal-enriched connections that were not observed in other groups after 6 months of treatment. This study provides the first detailed analysis of dysbiosis of the gut mycobiota due to active TB and anti-TB treatment. The results suggest that fungi play an important role in the balance of the gut microbiota and may be associated with the progression of TB, influencing the microbiota and immunity homeostasis in those receiving anti-TB treatment.

**IMPORTANCE** Numerous studies have shown that the gut bacterial microbiota is altered in active TB patients and that anti-TB drugs have profound and long-term impacts. However, as an integral part of the microbiota, fungi have rarely been studied. The need to investigate both the bacterial and fungal microbiota, as well as the relationship between them is apparent. The significance of our study is in our examination of the changes in the bacterial and fungal microbiota simultaneously in both active TB and patients receiving anti-TB treatment. We found that fungi play an important role in the bacterial-fungal transkingdom network, especially during the anti-TB therapy. These findings underscore the importance of fungi in gut microbiota dysbiosis during active TB and anti-TB treatment processes. In addition, our findings suggest it is meaningful to research potential adjunctive therapies that reduce fungal expansion and increase commensal bacterial abundance after anti-TB treatment, which would help the recovery of TB patients.

## INTRODUCTION

Tuberculosis remains the deadliest infectious disease, with a heavy burden worldwide and particularly in developing countries (1). The gut microbiome affects the homeostasis of immunity and provides resistance to invading pathogens (2–4), both in the intestine and in extraintestinal organs (5, 6). It has been demonstrated that in mice, disruption of the gut microbiota promotes susceptibility to *Mycobacteria tuberculosis* (MTB) infection (7). Furthermore, disruption of the gut microbiota also impairs the isoniazid (INH)-mediated MTB clearance by suppressing the innate immunity and reducing the CD4 T-cell response to MTB (8). It was found that the gut bacterial microbiome changed in the active TB patients compared with healthy subjects (9–12). Long-term anti-TB treatment, using four first-line anti-TB drugs, INH, rifampin (RIF), pyrazinamide (PZA), and ethambutol (EMB), induced a distinct and long-lasting dysbiosis of the gut bacterial microbiota (13). Members of the order Clostridiales were depleted, whereas taxa of the order Erysipelotrichales and Bacteroidales were enriched in the gut microbiota of TB patients during anti-TB treatment compared with healthy subjects (12–14).

The fungal microbiota, also known as the mycobiota, is an integral part of the gut microbiota ecosystem. Recent studies have revealed that the mycobiota also plays an important role in the homeostasis of host immunity (15, 16). The structure of the mycobiota is dynamic and responsive to host pathological changes in colorectal cancer, cirrhosis, inflammatory bowel disease (IBD), sepsis, and COVID-19 (17–21). Fungal members of the gut microbiota have been shown to influence the immune responses of the host by promoting or weakening inflammatory responses. For example, *Candida* species in the gut can regulate immune responses in the lungs by increasing plasma concentrations of prostaglandin-like immune response modulators, which induce pulmonary allergic responses (22, 23). Furthermore, it was found that gut commensal fungi can induce the antifungal IgG to protect the host against lethal systemic fungal infections (24). Therefore, the gut mycobiota contributes to the homeostasis of immunity, which might have influences on the progression of TB and the response to anti-TB treatment. It is also important to note that the prevalence of Candida albicans coinfection, which is associated with antibiotic exposure, is as high as 25.7% among TB patients in Asia and Africa (25, 26). However, previous studies in TB only focused on gut bacteria, and the role of fungi is unclear.

In this study, we investigated the gut bacterial microbiota and mycobiota of patients with active TB and those undergoing anti-TB treatment by 16S rRNA and ITS2 amplicon sequencing, respectively. Our results showed that the gut mycobiota was altered in both naive and anti-TB-treated TB patients, especially in those who received long-term anti-TB treatment. The *Nakaseomyces* and *Purpureocillium genera*, containing many opportunistic fungal pathogens, were enriched in TB patients. Moreover, a density decreased bacterial-fungal transkingdom network was found in the naive TB patients compared with healthy subjects. The transkingdom network was severely altered in patients after 2 months of anti-TB treatment and it had new connections that were not observed in other groups characterized by fungal enrichment after 6 months of treatment. The findings of this work indicated that fungi play an important role in the homeostasis of the gut microbiota, and they may be associated with the variable pathogenesis observed in MTB-infected individuals.

## RESULTS

### Bacterial microbiota of naive and anti-TB-treated TB patients exhibit reduced diversity and altered composition compared with healthy subjects.

**FIG 1:**
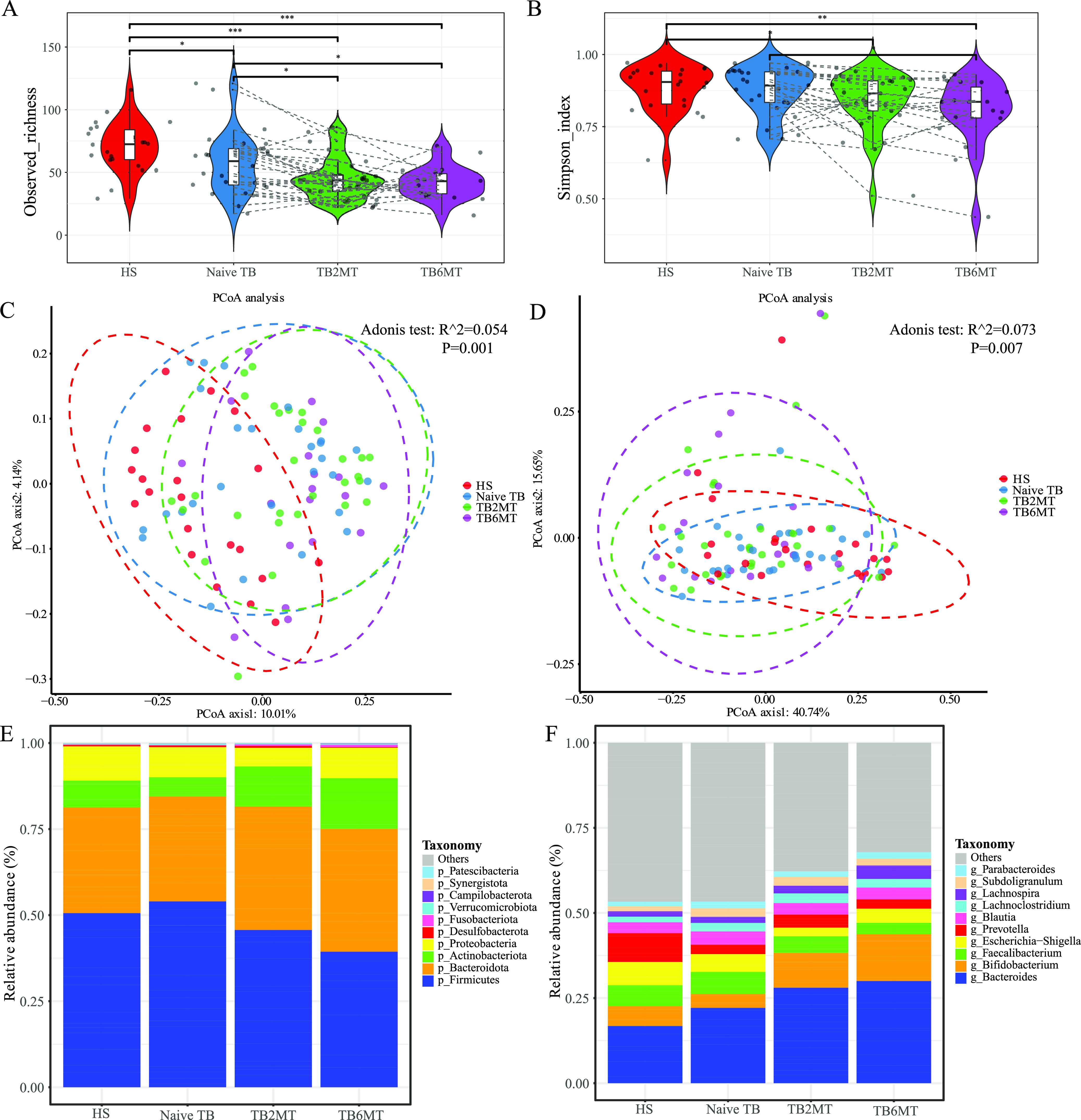
Altered bacterial microbiota biodiversity in TB patients. The α-diversity of the bacterial microbiota at the genus level based on (A) observed richness and (B) the Simpson index. PCoA analysis based on (C) unweighted UniFrac distance and (D) weighted UniFrac distance. The dissimilarity was analyzed using Adonis tests with 999 permutations. R^2 value (effect size) which means the percentage of the variation explained by the grouping, as well as *P value* to determine the statistical significance were shown in the figures. Global composition of bacterial microbiota at the (E) phylum and (F) genus levels. Only the top 10 taxa were presented in the graph. HS, healthy subjects; Naive TB, naive TB patients; TB2MT, TB patients after 2 months of treatment; TB6MT, TB patients after 6 months of treatment. (Wilcoxon Rank Sum test, *, *P*  <  0.05; **, *P*  <  0.01; ***, *P*  <  0.001).

We performed sequencing of the V3-V4 hypervariable region of 16S rRNA to investigate the bacterial portion of the gut microbiota. In summary, the sequences of V3-V4 region were successfully assigned to 355 specific genera across all samples, and 102 of them were shared among the four groups (Fig. S1A). The α-diversity, estimated by observed genus richness, showed that the diversity was significantly decreased in treatment-naive TB patients (*p* < 0.05) (Fig. 1A). TB patients after 2 months of treatment (TB2MT) and TB patients after 6 months of treatment (TB6MT) also showed reduced diversity compared to both the healthy subjects (HS) group and the naive TB group (Fig. 1A and B), which indicated that the gut bacterial community was considerably impacted by anti-TB drugs. The β diversity analyses were conducted on the basis of weighted and unweighted UniFrac distances, separately. The differences observed among the four groups were statistically significant (Fig. 1C and D). In particular, healthy subjects and all TB groups showed statistically significant differences based on unweighted and weighted UniFrac distances.

In line with previous studies, the bacterial microbiota was dominated by species from the Firmicutes, Bacteroidetes, Actinobacteria, and Proteobacteria phyla (Fig. 1E). In TB patients undergoing anti-TB treatment, the average relative abundance of the Firmicutes was decreased, while Bacteroidetes and Actinobacteria were increased (Fig. 1E). At the genus level, we observed that the average relative abundance of *Bacteroides* was gradually increased in HS, naive TB, TB2MT, and TB6MT. The abundance of *Bifidobacterium* was dramatically expanded in patients undergoing anti-TB drug treatment, and *Prevotella* was reduced in all TB groups (Fig. 1F).

### Mycobiota diversity analysis indicates fungal dysbiosis in naive and anti-TB treatment TB patients.

**FIG 2:**
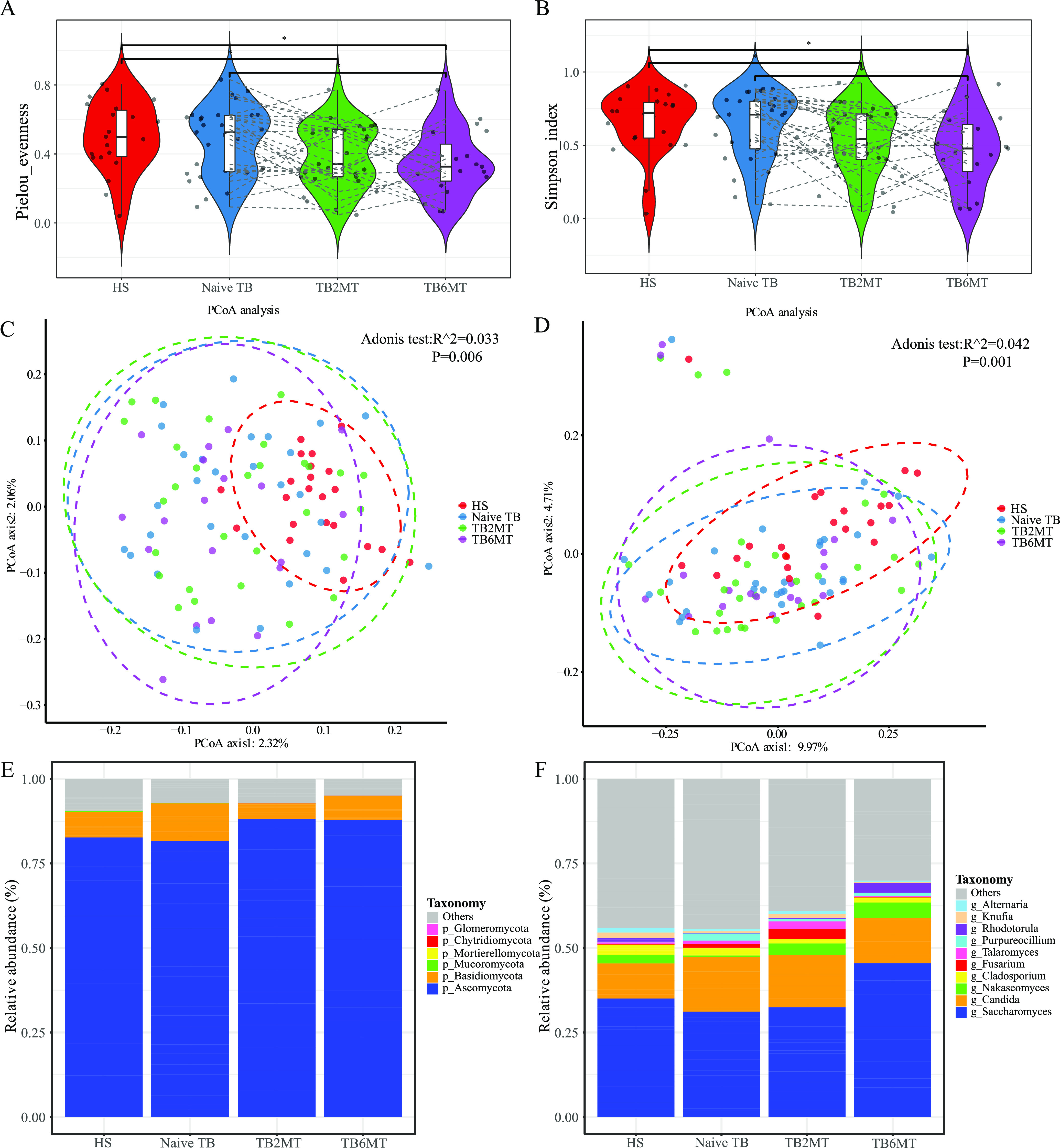
Altered mycobiota biodiversity in TB patients. The α-diversity of the mycobiota at the genus level based on (A) Pielou evenness and (B) the Simpson index. PCoA analysis using the (C) Jaccard distance matrix and (D) unweighted UniFrac distance. The dissimilarity was analyzed using Adonis tests with 999 permutations. R^2 value (effect size) which means the percentage of the variation explained by the grouping, as well as *P value* to determine the statistical significance were shown in the figures. Global composition of the mycobiota at the (E) phylum and (F) genus levels among four study groups. Only the top 10 taxa were presented in the graph. HS, healthy subjects; Naive TB, naive TB patients; TB2MT, TB patients after 2 months of treatment; TB6MT, TB patients after 6 months of treatment. (Wilcoxon Rank Sum test, *, *P*  <  0.05).

Using ITS2 sequencing, we estimated the composition and diversity of the mycobiota among the four groups in this study. In total, the ITS2 sequences were successfully assigned to 348 certain genera across all samples, and 83 of them were shared among the four groups (Fig. S1B). The α-diversity analyses showed that compared with HS group, the Pielou evenness and Simpson index were significantly decreased in the TB2MT and TB6MT groups, respectively. However, no obvious difference was observed between the HS and the naive TB groups (Fig. 2A and B). Compared with the naive TB group, these indexes were decreased to a level that approached significance in the TB2MT and were significantly decreased in the TB6MT group. The β-diversity analysis, based on Jaccard distance and unweighted UniFrac distance, showed that HS samples were distributed in a narrow area, while the TB patient samples had a dispersed distribution (Fig. 2C and D). There were statistically significant differences among the four groups. In particular, there were significant differences between the healthy subjects and naive TB patients.

Structural analysis of the mycobiota showed that the Ascomycota and Basidiomycota phyla were the dominant taxa in the four groups. Over 80% of fungi in all groups belonged to the Ascomycota, and their average relative abundance increased in the anti-TB groups (Fig. 2E). At the genus level, *Saccharomyces* and *Candida* were the most abundant taxa. The average relative abundance of the top 10 genera increased with the time of anti-TB treatment. Specifically, the abundance of *Saccharomyces* was lower in the naive TB and TB2MT groups, and higher in the TB6MT group. The abundance of *Candida* expanded in the three TB patient groups when compared with the healthy subject group (Fig. 2F).

### The bacterial and fungal microbiota compositions are altered in naive and anti-TB treatment TB patients.

**FIG 3:**
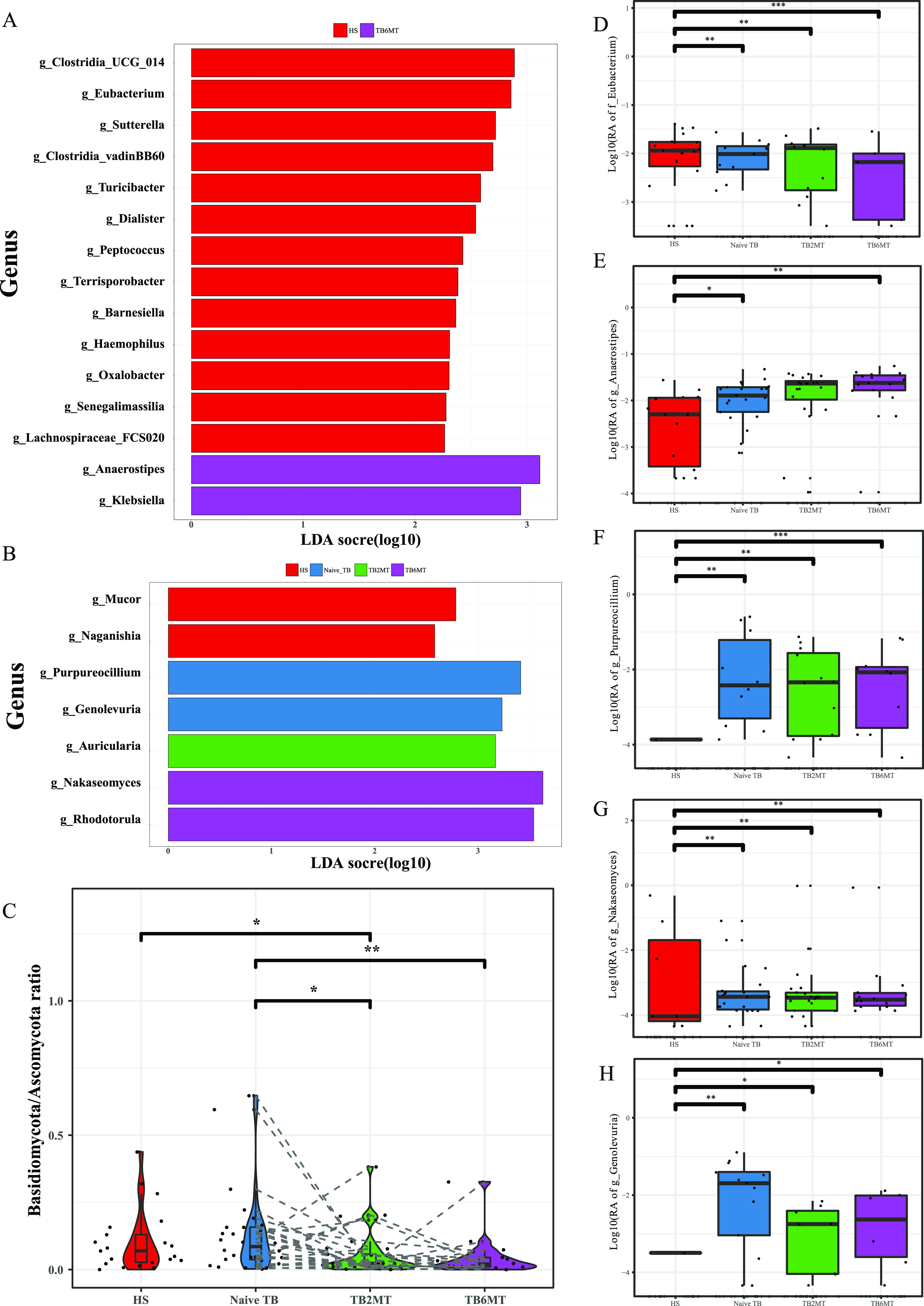
Characteristics of the bacterial and fungal taxa in the four study groups. Specific changes of (A) bacterial and (B) fungal relative abundance (RA) analyses using linear discriminant analysis effect size (LEfSe). Only genera with an LDA score (log_10_) of  > 2.0 were included. (C) Ratio of Basidiomycota/Ascomycota abundance in the four study groups. The RAs of (D) *Eubacterium*, (E) *Anaerostipes*, (F) *Purpureocillium*, (G) *Nakaseomyces*, and (H) *Genolevuria* were compared among the four study groups, separately. HS, healthy subjects; Naive TB, naive TB patients; TB2MT, TB patients after 2 months of treatment; TB6MT, TB patients after 6 months of treatment. (Wilcoxon Rank Sum test, *, *P*  <  0.05; **, *P*  <  0.01; ***, *P*  <  0.001).

To determine the taxa that associated with gut microbiota dysbiosis in the naive and anti-TB-treated TB patients, linear discriminant analysis effect size (LEfSe) was used to analyze the different abundances of bacteria and fungi among the groups. The results indicated that most of the differentially abundant bacterial taxa were enriched in healthy subjects group, mainly including the taxa of classes Clostridiales (including genera *Clostridia* UCG_014, *Clostridia* vadinBB60, *Lachnospiraceae* FCS020, *Eubacterium*, *Ruminococcus*, *Peptococcus*, and *Terrisporobacter*), Bacteroidia (including genera *Bacteroides*, *Barnesiella*, and *Alistipes*) and Gammaproteobacteria (including genera *Oxalobacter*, *Sutterella*, and Haemophilus) (Fig. 3A, Fig. S2). Among these, the species of order Oscillospirales, including species Eubacterium coprostanoligenes and Ruminococcus callidus, were enriched in HS and Naive TB group, and showed a tendency to decrease in TB2MT and TB6MT groups (Fig. S2A, C). The genera Anaerostipes and Klebsiella had a low abundance in HS and Naive TB, but showed a tendency to increase in the TB2MT and TB6MT groups (Fig. 3A and E, Fig. S2). For the fungi, we found that the genera *Naganishia* and *Mucor* were enriched in the HS group but depleted in the three TB groups (Fig. 3B). The genera Purpureocillium (mainly including Purpureocillium lilacinum) and Genolevuria (mainly including Genolevuria amylolytica) were depleted in HS group but enriched in naive TB group, and showed a tendency to decrease in anti-TB treatment (Fig. 3B, F, and H, Fig. S2). Moreover, the genera Nakaseomyces and Rhodotorula had low abundance in HS group, yet enriched in the TB groups and showed a tendency to increase with the time of anti-TB treatment (Fig. 3B and G, Fig. S2). In addition, we explored the changes in the rates of the two major fungal phyla, the Basidiomycota and Ascomycota. The results showed that long-term anti-TB drug treatment considerably affected the Basidiomycota/Ascomycota ratio. Specifically, compared with the HS or naive TB group, the Basidiomycota/Ascomycota ratios of both the TB2MT and TB6MT groups were significantly decreased (Fig. 3C).

### The transkingdom network is skewed in naive TB patients and distorted in long-term anti-TB treatment patients.

**FIG 4:**
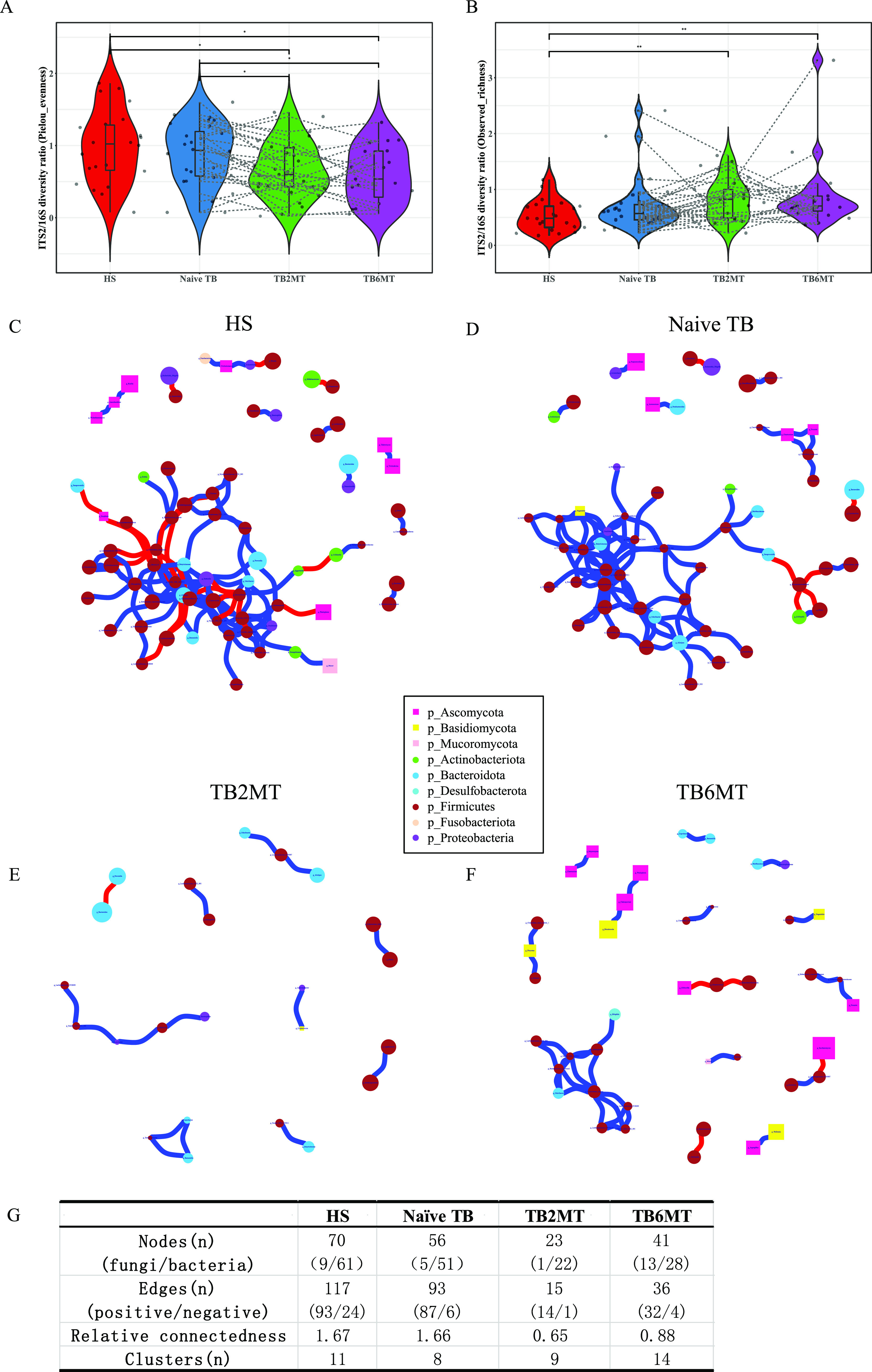
Fungal-bacterial equilibration analyses in the four study groups. The ITS2/16S diversity ratio was calculated by the Pielou evenness at the (A) order level and by observed richness at the (B) genus level (Wilcoxon Rank Sum test, *, *P*  < 0.05; **, *P*  <  0.01). Transkingdom abundance correlation networks of (C) HS, (D) naive TB, (E) TB2MT, and (F) TB6MT groups at the genus level are shown using the R package igraph. Each node represents a genus, and its color represents the phylum to which it belongs. The shape of the nodes represents the kingdom to which they belong. Squares represent fungi and circles represent bacteria. Node size represents the mean abundance of each genus. Edges indicate the magnitude of correlations (positive in blue and negative in red). Only genera presented in >30% of the samples in at least one group were considered, and only significant correlations with *P*  ≤  0.10 after false discovery rate correction and (|r| ≥0.6 are displayed). (G) The parameters of the networks of each group. The relative connectedness of the network was calculated as the ratio of edges (the number of significant interactions) and nodes (the number of genera). HS, healthy subjects; Naive TB, naive TB patients; TB2MT, TB patients after 2 months of treatment; TB6MT, TB patients after 6 months of treatment. (Wilcoxon Rank Sum test, *, *P*  <  0.05; **, *P*  <  0.01).

To understand the balance in the bacterial and fungal diversity, we analyzed the ITS2/16S diversity ratio based on Pielou evenness and observed richness indexes, separately. Compared with the HS or naive TB groups, the ITS2/16S Pielou evenness ratio of anti-TB treatment groups (TB2MT, TB6MT) was significantly decreased, while the ITS2/16S observed richness diversity ratio tended to increase with the time of anti-TB treatment (Fig. 4A and B). These results indicated that after administration of anti-TB drugs, the fungal richness increased and the bacterial richness decreased in TB patients.

To explore the potential interplay between bacteria and fungi, transkingdom abundance correlation network analyses were performed at the genus level in the HS, naive TB, TB2MT, and TB6MT groups, respectively. The results showed that the healthy subject group had the most complex network, in which 87.14% of nodes were bacterial nodes (Fig. 4C and G, Table S4). In the naive TB patient group, the complexity of the network was reduced, which suggested that the homeostasis in the gut microbiota was unbalanced during active TB (Fig. 4D, Table S1). Remarkably, the network of the TB2MT group was severely altered. Only the dyads, triads, quadruplets, and quintuplets composed of 23 genera were found in the network (Fig. 4E and G, Table S2). In the TB6MT group, there was a partial restoration of the network (Fig. 4G). However, the proportion of fungal nodes in the network was noticeably increased compared with the other three groups (Fig. 4G). Moreover, many dyads, triads, quadruplets, and quintuplets on had no connection with the major connected network (Fig. 4F and G, Table S3). Furthermore, negative relationships were enriched in the HS correlation network but rare in the other three TB groups (Fig. 4G). These results indicated that active TB was involved in an alteration in the bacterial-fungal transkingdom interactions, and the ecological balance of the gut microbiota is considerably affected by the anti-TB drugs.

## DISCUSSION

In this study, we showed that the diversity of the gut bacterial microbiota was decreased in naive TB patient group compared with healthy subject group, which agrees with previous studies (9, 27). Of the anti-TB drugs used in this study, only RIF has broad-spectrum antibacterial activities. INH, PZA, and EMB are specifically targeted to mycobacterial species (28). These drugs notably altered the diversity of the gut bacterial microbiota. Both in phyla and genus level, the composition of bacterial microbiota has changed in naive-TB group compared with HS group, but showed distinct shifts in the TB2MT and TB6MT groups (Fig. 1E and F). These results suggested that the gut bacterial microbiota was disordered in treatment-naive TB patients and that anti-TB treatment had a further influence on it. According to the differential abundance analysis, we found that the alteration of the gut bacterial microbiota was mainly associated with the class *Clostridiales*, which has a vital role in gut microbiome homeostasis and immune balance, particularly via their metabolite short-chain fatty acids (SCFAs) (29). For example, the order *Oscillospirales*, including Eubacterium coprostanoligenes and Ruminococcus callidus, was found enriched in the HS and naive TB groups, and significantly decreased in the anti-TB treatment groups (Fig. 3D, Fig. S2C). However, the genus, Anaerostipes, was considerably enriched in the naive TB and anti-TB treatment groups (Fig. 3E). It has been reported that the main species, Anaerostipes hadrus BPB5, has no detrimental effect in healthy mice, but can aggravate accelerated dysbiosis of gut microbiota in mice with colitis (30). This result was compatible with the previous opinion that some butyrate-producing bacteria enriched in TB patients may have negative effects on their health (11, 31). In addition, the genus Klebsiella, which is dominated by pathobionts, was found enriched in the naive TB groups, and this might represent an imbalance of the gut microbiota.

Most notably, this is the first study to our knowledge to investigate the gut mycobiota dysbiosis in TB. There was no difference in the α-diversity of the mycobiota between healthy subjects group and naive TB patients group, but PCoA analysis showed a significant difference between them. This suggested that gut fungi may also have influence the progression of active TB. The anti-TB drug usage led to a significant decrease in the biodiversity of the gut mycobiota (Fig. 2A and B). This result indicated that the structure of the gut mycobiota was also impacted by taking the first-line anti-TB drugs. Consistent with other studies, the Ascomycota and Basidiomycota were the most abundant phyla in the gut mycobiota. Changes in their ratio (Basidiomycota/Ascomycota) may be involved in the inflammatory process (32); it was significantly reduced in patients undergoing anti-TB treatment compared with healthy subjects and naive TB patients. Differential abundance analysis found that some pathogenic genera/species were enriched in TB patients. For example, Purpureocillium lilacinum, an emerging pathogenic species that can cause pulmonary, ocular, and cutaneous and/or subcutaneous infections (33), was not detected in almost all healthy subjects, but enriched in the naive TB group. Its relative abundance was decreased with the time of anti-TB drug treatment (Fig. 3F, Fig. S2H). The relative abundance of genus *Nakaseomyces* (Fig. 3G), including six closely related species, three of which are pathogenic (34), was variable in healthy subjects but low in most of them. It was found enriched in TB6MT group compared with HS group, and its relative abundance was increased with the time of anti-TB drug treatment.

Bacteria and fungi live together in the gut and occupy the same ecological niche. They will inevitably interact with each other. The α-diversity ratio of ITS2/16S could reflect the structural alterations in the fungal-bacterial microbiota (19, 32, 35); our results showed that it was strongly impacted by anti-TB drugs. In addition, transkingdom abundance correlation network analysis was performed to investigate the balance in the gut microbiota. We found that the network was obviously simplified in the naive-treatment active TB patients. Homeostasis of the gut microbiota generally represents a good immune state (36). This result suggested that the gut microbiota plays a crucial role in the progression of active tuberculosis. Strikingly, the bacterial-fungal correlation network was severely impacted after 2 months of anti-TB treatment. This result indicated that four first-line anti-TB drugs had a profound effect on the structure of the gut microbiota. Dysbiosis can be characterized as a loss of beneficial bacteria and an overgrowth of fungi (37, 38). As we can see in the TB6MT group, the network structure was rebuilt in a different form in which fungi occupied an important position in the network. This characteristic most likely indicates that the gut microbiota is in a state of disorder and an increase in the risk of PTB-fungal co-infection duration anti-TB drug treatment. For those TB patients undergoing anti-TB treatment, the gut bacterial microbiota dysbiosis can persist for at least 1.2 years (13), and it has been demonstrated that gut microbiota dysbiosis can compromise alveolar macrophage immunity to MTB, provoke susceptibility to TB and limit the INH mediated clearance of MTB in mice (7, 8, 39). Recently, Jain et al. discovered that antibiotic treatment could result in overgrowth of the fungi during the gut mucosal injury and can impair mucosal healing (40). It is possible that anti-TB treatment has a long-lasting effect on the homeostasis of the gut microbiota, which may have influences on the health of those cured subjects.

As a whole, the microbiota is composed of bacteria, fungi, viruses, and protozoa. Inhabiting the intestinal lumen and mucosa, they obtain nutrients that are required for their proliferation and colonization from other microorganisms, and they develop intricate ecological networks through these interactions (41). The homeostasis of the microbiota ecological network protects us from dysbiosis-related disease (42). In general, antibiotics are specific to the bacterial community. However, as the most important part of the microbiota, any alteration in the bacterial structure inadvertently leads to changes in the fungal community (18, 43, 44). Emerging evidence has suggested that targeting fungi can also lead to alterations in the bacterial community. For example, oral treatment of mice with antifungal drugs exhibited noticeable structural alterations in the bacterial community (45). Colonization by five yeast species could induce strong ecological changes in the gut bacterial microbiota in gnotobiotic mice (46). Taken together, transkingdom alterations can lead to transkingdom dysbiosis, which can then have an influence on the development and regulation of the immune system, increasing the risk of infectious disease (42) (Fig. S3). Fungal dysbiosis has also been associated with reduced efficacy of fecal microbiota transplantation (FMT) in Clostridium difficile infection (47). Therefore, it would be beneficial to recover the homeostasis of the gut microbiota after anti-TB treatment. Furthermore, it is necessary to conduct follow-up studies on the effectiveness and necessity of taking probiotics, prebiotics, or by undergoing FMT after treatment for tuberculosis patients. Moreover, investigating how transkingdom dysbiosis impacts on immunity and health and how long it lasts after completing anti-TB treatment is worthy of further research.

There were some limitations in this study. First, the gender and age of the healthy subject group was not very closely matched with the TB patients, which may have adversely affected the accuracy of the results when the HS group was compared with the other groups. However, the major findings of gut mycobiota dysbiosis in follow-up TB patients were unaffected. Second, there were 10 patients lost to follow-up in the TB6MT because of the influences of the COVID-19 epidemic and other causes. Yet most of them were missing at random, and there were no difference in clinical characteristics between TB6MT and naive-TB/TB2MT groups. Therefore, the impact of patients lost to follow-up to the accuracy of results is limited. Third, the long-term effects of mycobiota dysbiosis after completion of therapy were not investigated in this study. Future work should therefore include long-term follow-up after treatment with a large-scale population to evaluate the influence of microbiota dysbiosis on health.

## CONCLUSIONS

In summary, this is the first study to investigate gut mycobiota dysbiosis in naive and anti-TB treatment TB patients. Our results demonstrated that both bacteria and fungi experienced dysbiosis in the naive-treatment TB patients. Long-term anti-TB drug usage severely altered both the bacterial and fungal gut microbiotas. In particular, the order Oscillospirales, including Eubacterium coprostanoligenes and Ruminococcus callidus, was found enriched in the healthy subjects, and its abundance showed a tendency to decrease in the anti-TB treatment groups. Moreover, the abundance of *Purpureocillium* tended to decrease and *Nakaseomyces* tended to increase during anti-TB treatment, and both had increased proportions in the three TB groups compared with healthy subjects. We also found that there was an altered transkingdom network in naive TB patients. The network was greatly diminished in patients after 2 months of anti-TB treatment; and it was rebuilt in a dysbiotic state characterized by an enrichment in fungal nodes after 6 months of treatment.

## MATERIALS AND METHODS

### Study cohort and sample collection.

We recruited 29 naive active pulmonary TB patients and 22 healthy subjects from Beijing Changping Institute for Tuberculosis Prevention and Treatment, and Shijiazhuang Gaocheng People’s Hospital. The TB patients were diagnosed according to clinical symptoms based on *The National Guidelines for the Diagnosis of Pulmonary Tuberculosis (WS 288—2017)* and *The Classification of Tuberculosis (WS 196—2017)* (48, 49), including the results of the T-SPOT.TB test, MTB sputum culture, microscopic examination of sputum smears, and chest radiograph. For the 29 TB patients, 17 of them were microbiologically confirmed and 12 of them were clinically confirmed. All 29 TB patients received 6 months of standard anti-TB therapy, taking INH, RIF, EMB, and PZA in the first 2 months and INH and RIF in the following 4 months. And they were advised to increase their intake of proteins, vegetables, and fruits. Fecal samples were collected at baseline (29 samples), 2 months (29 samples), and 6 months (19 samples). Because of the influences of the COVID-19 epidemic and other causes, 10 patients of TB6MT group were lost to follow-up in the 6 months. All 19 TB patients who completed the 6-month anti-TB treatment recovered after the treatment. Samples were immediately refrigerated and then stored at −80°C for further analysis. Individuals who had other human microbiome-associated diseases (including diabetes, obesity, gastroenteritis, and HIV infection) not related to TB and who administered any antibiotics before 2 months of sampling were excluded. The characteristics of the individuals included in this study are depicted in Table 1. The study protocol was approved by the ethics committee of the Institute of Microbiology, Chinese Academy of Science (no. APIMCAS2019051). Informed consent was obtained from all the study participants.

**TABLE 1:** Characteristics of individuals in the study groups^*a*^

Characteristic	HS (*N* = 22)^*b*^	Naive TB (*N* = 29)^*b*^	TB2MT (*N* = 29)^*b*^	TB6MT (*N* = 19)^*b*^	*P*-value^*c*^
Gender					0.2
female	4 (18%)	11 (38%)	11 (38%)	9 (47%)	
male	18 (82%)	18 (62%)	18 (62%)	10 (53%)	
Age	50.00 (34.25, 58.50)	36.00 (27.00, 47.00)	36.00 (27.00, 47.00)	36.00 (28.50, 46.50)	0.10
BMI	22.41 (19.72, 24.41)	20.83 (18.93, 22.89)	20.83 (18.93, 22.89)	21.34 (19.41, 22.75)	0.2

a HS, healthy subjects; Naive TB, naive TB patients; TB2MT, TB patients after 2 months of treatment; TB6MT, TB patients after 6 months of treatment.

b n (%); Median (IQR).

c Pearson’s chi-squared test; Kruskal-Wallis rank-sum test.

### DNA extraction and sequencing.

DNA was extracted from 200 mg of feces using the QIAamp DNA stool minikit. The V3-V4 region of bacterial 16S rRNA and the ITS2 region of fungi, which contain the high nucleotide heterogeneity and display the high discriminatory power at multiple taxonomic ranks, are widely used in the studies of various microbiota (50, 51). The V3-V4 regions of the bacterial 16S rRNA genes were amplified from each DNA sample using the F3 (5′-CCTACGGGNBGCASCAG-3′) and R4 (5′-GACTACNVGGGTATCTAATCC-3′) primers. The ITS2 regions of the fungal 18S-26S ribosomal DNA cistron were amplified with ITS-V2-F (5′-GTGARTCATCGAATCTTT-3′) and ITS-V2-R (5′-GATATGCTTAAGTTCAGCGGGT-3′) primers. The sequencing libraries were constructed following the procedures recommended by the manufacturer and then sequenced in 250 bp paired end (PE-250) runs using the Illumina HiSeq 2500 platform.

### Bioinformatics analysis.

The raw data were demultiplexed using the qiime2-2019.7 packages. Amplicon sequence variants (ASVs) were created using DADA2 by grouping unique sequences (52). Taxonomic composition analysis was explored using the q2-feature-classifier plugin (qiime2) with Naïve Bayes Classifiers trained by us (Silva reference database version-138 for bacteria and UNITE ITS database version-8.2 for fungi) (53, 54). Rarefaction was performed (9,326 sequences per sample for 16S and 21,916 sequences for ITS2) for downstream analysis. The α-diversity at the genus level was estimated by the observed richness index, Pielou evenness index, ACE index, and Simpson index, respectively. The β-diversity analysis was conducted using weighted/unweighted UniFrac distance and Jaccard distance matrix. Sequencing data are available in the NCBI (SRA) (accession number PRJNA715947).

### Statistical analyses.

R3.6.3 was used for downstream analysis and graph generation. For graphical data, statistical analyses were performed using the Wilcoxon rank sum test or the Kruskal–Wallis test with Dunn’s multiple comparison test under suitable situations. The α- and β-diversity analyses were carried out using the vegan package, version 2.5–7 (55). Statistical significance analyses were performed using permutational multivariate analysis of variance tests (PERMANOVA/Adonis) in PCoA analyses. Plots were drawn using the ggplot2 package. Linear discriminant analysis effect size (LEfSe) was used to identify changes in bacterial and fungal abundances between groups (56). Correlation analyses were performed using the Spearman method, and the *P values* were corrected to control the false discovery rate using the package psych, version 2.0.12. Transkingdom network figures were built using the package igraph, version 1.2.6.
