# Supplementary material for: Gut Mycobiota Dysbiosis in Pulmonary Tuberculosis Patients Undergoing Anti-Tuberculosis Treatment
Source: Microbiol Spectr. 2021 Dec 15;9(3):e00615-21. doi: 10.1128/spectrum.00615-21 (PMC8672887; doi:10.1128/spectrum.00615-21)
Supplement: SUPPLEMENTAL FILE 1 — Supplemental material. Download SPECTRUM00615-21_Supp_1_seq2.pdf, PDF file, 0.4 MB [file spectrum00615-21_supp_1_seq2.pdf]

Supplementary figures

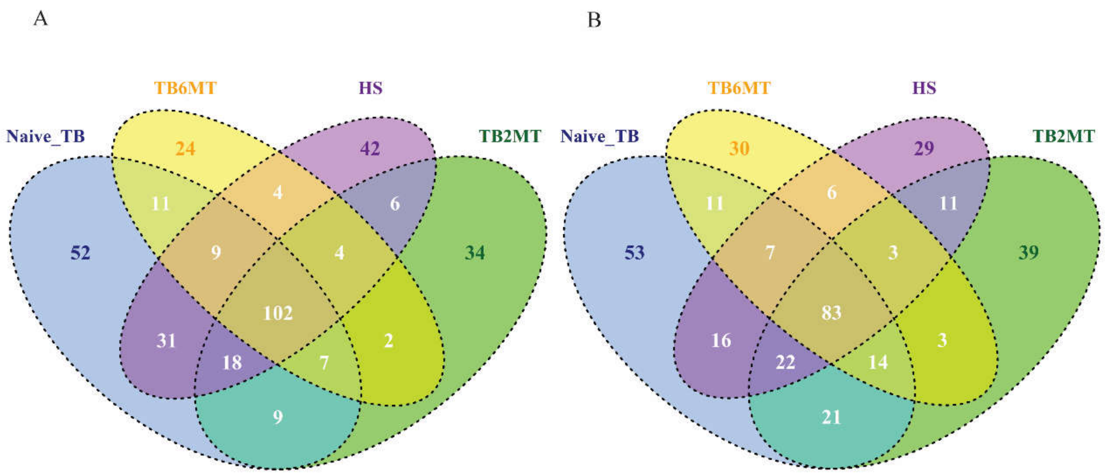

Figure S1 Genus composition analyses of the bacterial and fungal microbiota among the four groups. (A) Composition analysis of the bacterial genera within the gut bacterial microbiota. (B) Composition analysis of the fungal genera within the gut mycobiota. HS: healthy subjects; Naive TB: naive TB patients; TB2MT: TB patients after 2 months of treatment; TB6MT: TB patients after 6 months of treatment.

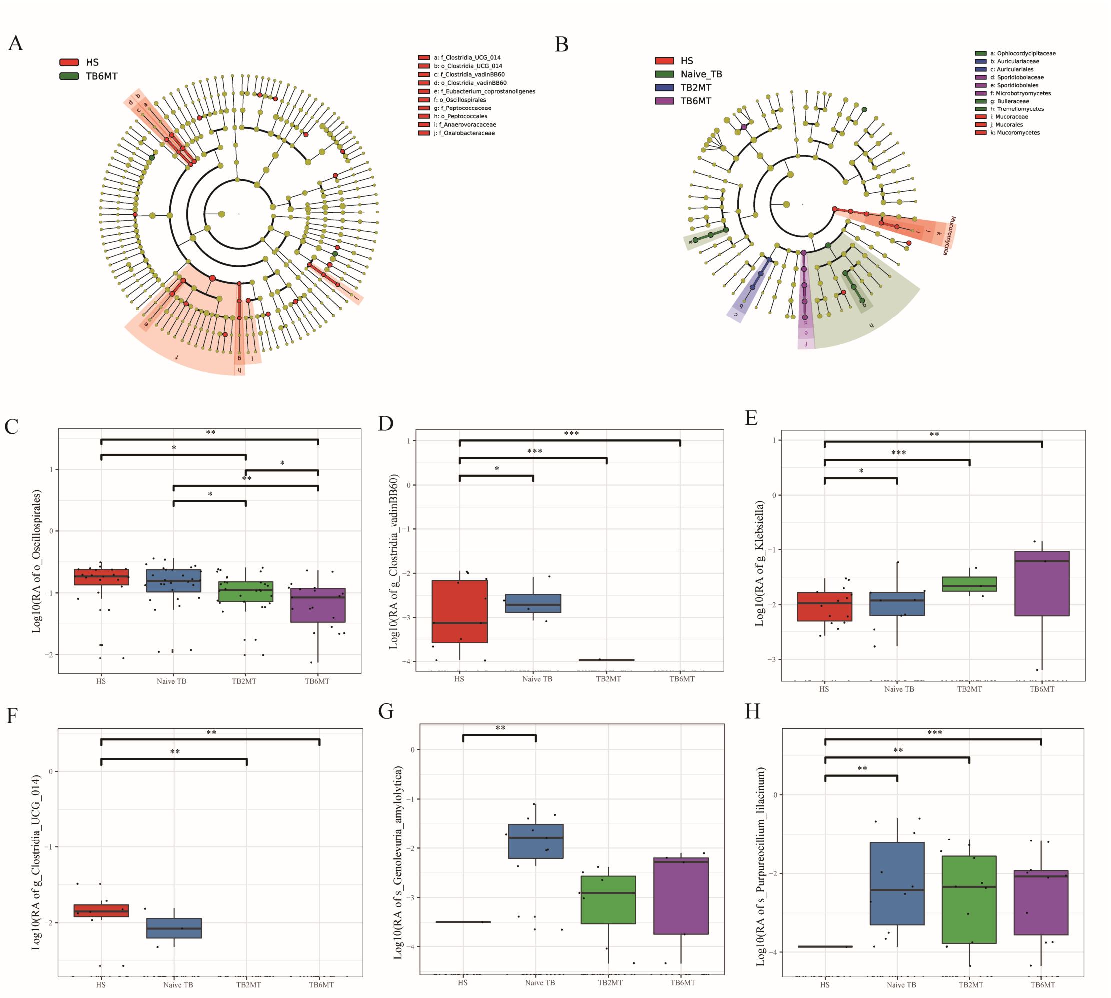

Figure S2 Analysis of differential taxa abunsances among the four groups. Significantly different taxa of bacteria (A) and fungi (B) shown in the cladogram identified by LEfSe. The relative abundances (RAs) of *Eubacterium coprostanoligenes* (C), *Clostridia vadinBB60* (D), *Klebsiella* (E), *Clostridia UCG_014* (F), *Genolevuria*

amylolytica (G) and *Purpureocillium lilacinum* (H) were compared among the four groups. HS: healthy subjects; Naive TB: naive TB patients; TB2MT: TB patients after 2 months of treatment; TB6MT: TB patients after 6 months of treatment (Wilcoxon Rank Sum test, *p<0.05; **p<0.01; ***p<0.001)
