# Supplementary material for: Gut Mycobiota Dysbiosis in Pulmonary Tuberculosis Patients Undergoing Anti-Tuberculosis Treatment
Source: Microbiol Spectr. 2021 Dec 15;9(3):e00615-21. doi: 10.1128/spectrum.00615-21 (PMC8672887; doi:10.1128/spectrum.00615-21)
Supplement: SUPPLEMENTAL FILE 2 — Supplemental material. Download SPECTRUM00615-21_Supp_2_seq3.pdf, PDF file, 0.2 MB [file spectrum00615-21_supp_2_seq3.pdf]

**Table S1** The results of Spearman abundance correlation analysis of naive TB group at the genus level. Only significant correlations with p⩽ 0.10 after false discovery rate correction and |r| ⩾ 0.6 are displayed.

| **genus_a** | **genus_b** | **r** | **p value** | **p(FDR)** |
|---|---|---|---|---|
| k_Fungi;p_Ascomycota;c_Sordariomycetes;o_Sordariales;f_Chaetomiaceae;g_Chaetomium | k_Fungi;p_Ascomycota;c_Dothideomycetes;o_Pleosporales;f_Sporormiaceae;g_Preussia | 0.841714438 | 1.99E-08 | 6.63E-05 |
| k_Bacteria;p_Firmicutes;c_Bacilli;o_Erysipelotrichales;f_Erysipelotrichaceae;g_Clostridium_innocuum | k_Fungi;p_Ascomycota;c_Dothideomycetes;o_Pleosporales;f_Sporormiaceae;g_Preussia | 0.691135301 | 4.66E-05 | 0.013841455 |
| k_Bacteria;p_Bacteroidota;c_Bacteroidia;o_Bacteroidales;f_Tannerellaceae;g_Parabacteroides | k_Fungi;p_Ascomycota;c_Sordariomycetes;o_Hypocreales;f_Hypocreales_fam_Incertae_sedis;g_Acremonium | 0.621044363 | 4.21E-04 | 0.053115464 |
| k_Bacteria;p_Proteobacteria;c_Gammaproteobacteria;o_Pseudomonadales;f_Moraxellaceae;g_Acinetobacter | k_Fungi;p_Ascomycota;c_Sordariomycetes;o_Hypocreales;f_Ophiocordycipitaceae;g_Purpureocillium | 0.612290503 | 5.34E-04 | 0.059911637 |
| k_Bacteria;p_Firmicutes;c_Bacilli;o_Erysipelotrichales;f_Erysipelotrichaceae;g_Clostridium_innocuum | k_Fungi;p_Ascomycota;c_Sordariomycetes;o_Sordariales;f_Chaetomiaceae;g_Chaetomium | 0.670917616 | 9.33E-05 | 0.019656126 |
| k_Bacteria;p_Firmicutes;c_Clostridia;o_Oscillospirales;f_Ruminococcaceae;g_Candidatus_Soleaferrea | k_Fungi;p_Ascomycota;c_Sordariomycetes;o_Sordariales;f_Chaetomiaceae;g_Chaetomium | 0.616646135 | 4.75E-04 | 0.057612038 |
| k_Bacteria;p_Bacteroidota;c_Bacteroidia;o_Bacteroidales;f_Marinifilaceae;g_Butyricimonas | k_Fungi;p_Basidiomycota;c_Tremellomycetes;o_Filobasidiales;f_Filobasidiaceae;g_Naganishia | 0.637157663 | 2.66E-04 | 0.039525623 |
| k_Bacteria;p_Firmicutes;c_Clostridia;o_Oscillospirales;f_Ruminococcaceae;g_Eubacterium_siraeum | k_Fungi;p_Basidiomycota;c_Tremellomycetes;o_Filobasidiales;f_Filobasidiaceae;g_Naganishia | 0.666543575 | 1.08E-04 | 0.020531352 |
| k_Bacteria;p_Firmicutes;c_Clostridia;o_Peptococcales;f_Peptococcaceae;g_Peptococcus | k_Fungi;p_Basidiomycota;c_Tremellomycetes;o_Filobasidiales;f_Filobasidiaceae;g_Naganishia | 0.679408031 | 7.01E-05 | 0.017857232 |
| k_Bacteria;p_Proteobacteria;c_Gammaproteobacteria;o_Burkholderiales;f_Oxalobacteraceae;g_Oxalobacter | k_Fungi;p_Basidiomycota;c_Tremellomycetes;o_Filobasidiales;f_Filobasidiaceae;g_Naganishia | 0.828715682 | 5.14E-08 | 9.17E-05 |
| k_Bacteria;p_Firmicutes;c_Bacilli;o_Lactobacillales;f_Streptococcaceae;g_Streptococcus | k_Bacteria;p_Actinobacteriota;c_Actinobacteria;o_Actinomycetales;f_Actinomycetaceae;g_Actinomyces | 0.678053565 | 7.34E-05 | 0.018087092 |

| | | | | |
|---|---|---|---|---|
| k_Bacteria;p_Firmicutes;c_Bacilli;o_Erysipelotrichales;f_Erysipelatoclostridiaceae;g_Erysipelatoclostridium | k_Bacteria;p_Actinobacteriota;c_Coriobacteriia;o_Coriobacteriales;f_Coriobacteriaceae;g_Collinsella | -0.672885288 | 8.74E-05 | 0.019462767 |
| k_Bacteria;p_Firmicutes;c_Clostridia;o_Lachnospirales;f_Lachnospiraceae;g_Dorea | k_Bacteria;p_Actinobacteriota;c_Coriobacteriia;o_Coriobacteriales;f_Coriobacteriaceae;g_Collinsella | 0.611765529 | 5.42E-04 | 0.059911637 |
| k_Bacteria;p_Firmicutes;c_Bacilli;o_Erysipelotrichales;f_Erysipelotrichaceae;g_Holdemanella | k_Bacteria;p_Actinobacteriota;c_Coriobacteriia;o_Coriobacteriales;f_Eggerthellaceae;g_Senegalimassilia | 0.881870578 | 5.63E-10 | 5.02E-06 |
| k_Bacteria;p_Firmicutes;c_Bacilli;o_Lactobacillales;f_Lactobacillaceae;g_Lactobacillus | k_Bacteria;p_Bacteroidota;c_Bacteroidia;o_Bacteroidales;f_Bacteroidaceae;g_Bacteroides | -0.620837673 | 4.23E-04 | 0.053115464 |
| k_Bacteria;p_Firmicutes;c_Clostridia;o_Christensenellales;f_Christensenellaceae;g_Christensenellaceae_R_7 | k_Bacteria;p_Bacteroidota;c_Bacteroidia;o_Bacteroidales;f_Marinifilaceae;g_Butyricimonas | 0.748570772 | 4.62E-06 | 0.002574383 |
| k_Bacteria;p_Firmicutes;c_Clostridia;o_Clostridia_vadinBB60;f_Clostridia_vadinBB60;g_Clostridia_vadinBB60 | k_Bacteria;p_Bacteroidota;c_Bacteroidia;o_Bacteroidales;f_Marinifilaceae;g_Butyricimonas | 0.643279274 | 2.22E-04 | 0.034123129 |
| k_Bacteria;p_Firmicutes;c_Clostridia;o_Lachnospirales;f_Lachnospiraceae;g_Eubacterium_oxidoreducens | k_Bacteria;p_Bacteroidota;c_Bacteroidia;o_Bacteroidales;f_Marinifilaceae;g_Butyricimonas | 0.693152306 | 4.34E-05 | 0.013570443 |
| k_Bacteria;p_Firmicutes;c_Clostridia;o_Lachnospirales;f_Lachnospiraceae;g_Lachnospiraceae_NC2004 | k_Bacteria;p_Bacteroidota;c_Bacteroidia;o_Bacteroidales;f_Marinifilaceae;g_Butyricimonas | 0.830049647 | 4.68E-08 | 9.17E-05 |
| k_Bacteria;p_Firmicutes;c_Clostridia;o_Lachnospirales;f_Lachnospiraceae;g_Lachnospiraceae_UCG_001 | k_Bacteria;p_Bacteroidota;c_Bacteroidia;o_Bacteroidales;f_Marinifilaceae;g_Butyricimonas | 0.749232369 | 4.49E-06 | 0.002574383 |
| k_Bacteria;p_Firmicutes;c_Clostridia;o_Oscillospirales;f_Ruminococcaceae;g_Eubacterium_siraeum | k_Bacteria;p_Bacteroidota;c_Bacteroidia;o_Bacteroidales;f_Marinifilaceae;g_Butyricimonas | 0.664704912 | 1.14E-04 | 0.020770399 |
| k_Bacteria;p_Firmicutes;c_Clostridia;o_Peptostreptococcales_Tissierellales;f_Anaerovoracaceae;g_Family_XIII_AD3011 | k_Bacteria;p_Bacteroidota;c_Bacteroidia;o_Bacteroidales;f_Marinifilaceae;g_Butyricimonas | 0.652214656 | 1.69E-04 | 0.028415762 |
| k_Bacteria;p_Proteobacteria;c_Gammaproteobacteria;o_Burkholderiales;f_Oxalobacteraceae;g_Oxalobacter | k_Bacteria;p_Bacteroidota;c_Bacteroidia;o_Bacteroidales;f_Marinifilaceae;g_Butyricimonas | 0.608259974 | 5.95E-04 | 0.061674054 |

| | | | | |
|---|---|---|---|---|
| k_Bacteria;p_Bacteroidota;c_Bacteroidia;o_Bacteroidales;f_Rikenellaceae;g_Alistipes | k_Bacteria;p_Bacteroidota;c_Bacteroidia;o_Bacteroidales;f_Marinifilaceae;g_Odoribacter | 0.840214102 | 2.23E-08 | 6.63E-05 |
| k_Bacteria;p_Firmicutes;c_Clostridia;o_Christensenellales;f_Christensenellaceae;g_Christensenellaceae_R_7 | k_Bacteria;p_Bacteroidota;c_Bacteroidia;o_Bacteroidales;f_Marinifilaceae;g_Odoribacter | 0.601735329 | 7.06E-04 | 0.068354388 |
| k_Bacteria;p_Firmicutes;c_Clostridia;o_Lachnospirales;f_Lachnospiraceae;g_Eubacterium_ventriosum | k_Bacteria;p_Bacteroidota;c_Bacteroidia;o_Bacteroidales;f_Marinifilaceae;g_Odoribacter | 0.615586422 | 4.89E-04 | 0.0580615 |
| k_Bacteria;p_Firmicutes;c_Clostridia;o_Oscillospirales;f_Eubacterium_coprostanoligenes;g_Eubacterium_coprostanoligenes | k_Bacteria;p_Bacteroidota;c_Bacteroidia;o_Bacteroidales;f_Marinifilaceae;g_Odoribacter | 0.670402081 | 9.49E-05 | 0.019656126 |
| k_Bacteria;p_Firmicutes;c_Clostridia;o_Oscillospirales;f_Ruminococcaceae;g_Ruminococcus | k_Bacteria;p_Bacteroidota;c_Bacteroidia;o_Bacteroidales;f_Marinifilaceae;g_Odoribacter | 0.633257896 | 2.98E-04 | 0.041497623 |
| k_Bacteria;p_Firmicutes;c_Clostridia;o_Peptostreptococcales_Tissierellales;f_Anaerovoracaceae;g_Family_XIII_AD3011 | k_Bacteria;p_Bacteroidota;c_Bacteroidia;o_Bacteroidales;f_Marinifilaceae;g_Odoribacter | 0.608043024 | 5.98E-04 | 0.061674054 |
| k_Bacteria;p_Firmicutes;c_Bacilli;o_Erysipelotrichales;f_Erysipelotrichaceae;g_Holdemanella | k_Bacteria;p_Bacteroidota;c_Bacteroidia;o_Bacteroidales;f_Muribaculaceae;g_Muribaculaceae | 0.663542153 | 1.19E-04 | 0.021131411 |
| k_Bacteria;p_Firmicutes;c_Bacilli;o_Erysipelotrichales;f_Erysipelatoclostridiaceae;g_Erysipelatoclostridium | k_Bacteria;p_Bacteroidota;c_Bacteroidia;o_Bacteroidales;f_Prevotellaceae;g_Paraprevotella | -0.618332908 | 4.53E-04 | 0.056115434 |
| k_Bacteria;p_Firmicutes;c_Bacilli;o_Erysipelotrichales;f_Erysipelotrichaceae;g_Holdemanella | k_Bacteria;p_Bacteroidota;c_Bacteroidia;o_Bacteroidales;f_Prevotellaceae;g_Paraprevotella | 0.611263074 | 5.49E-04 | 0.059911637 |
| k_Bacteria;p_Firmicutes;c_Clostridia;o_Lachnospirales;f_Lachnospiraceae;g_Eubacterium_ventriosum | k_Bacteria;p_Bacteroidota;c_Bacteroidia;o_Bacteroidales;f_Rikenellaceae;g_Alistipes | 0.629278194 | 3.34E-04 | 0.045098296 |
| k_Bacteria;p_Firmicutes;c_Clostridia;o_Lachnospirales;f_Lachnospiraceae;g_Lachnospiraceae_ND3007 | k_Bacteria;p_Bacteroidota;c_Bacteroidia;o_Bacteroidales;f_Rikenellaceae;g_Alistipes | 0.633489194 | 2.96E-04 | 0.041497623 |
| k_Bacteria;p_Firmicutes;c_Clostridia;o_Lachnospirales;f_Lachnospiraceae;g_Lachnospiraceae_UCG_010 | k_Bacteria;p_Bacteroidota;c_Bacteroidia;o_Bacteroidales;f_Rikenellaceae;g_Alistipes | 0.631089338 | 3.17E-04 | 0.043485792 |

| | | | | |
|---|---|---|---|---|
| k_Bacteria;p_Firmicutes;c_Clostridia;o_Oscillospirales;f_Eubacterium_coprostanoligenes;g_Eubacterium_coprostanoligenes | k_Bacteria;p_Bacteroidota;c_Bacteroidia;o_Bacteroidales;f_Rikenellaceae;g_Alistipes | 0.607527022 | 6.06E-04 | 0.061674054 |
| k_Bacteria;p_Firmicutes;c_Clostridia;o_Oscillospirales;f_Oscillospiraceae;g_NK4A214 | k_Bacteria;p_Bacteroidota;c_Bacteroidia;o_Bacteroidales;f_Rikenellaceae;g_Alistipes | 0.616366025 | 4.78E-04 | 0.057612038 |
| k_Bacteria;p_Firmicutes;c_Clostridia;o_Oscillospirales;f_Ruminococcaceae;g_Ruminococcus | k_Bacteria;p_Bacteroidota;c_Bacteroidia;o_Bacteroidales;f_Rikenellaceae;g_Alistipes | 0.704837492 | 2.82E-05 | 0.009665989 |
| k_Bacteria;p_Firmicutes;c_Clostridia;o_Lachnospirales;f_Lachnospiraceae;g_Dorea | k_Bacteria;p_Firmicutes;c_Bacilli;o_Erysipelotrichales;f_Erysipelatoclostridiaceae;g_Erysipelatoclostridium | -0.602881778 | 6.85E-04 | 0.067363391 |
| k_Bacteria;p_Firmicutes;c_Clostridia;o_Lachnospirales;f_Lachnospiraceae;g_Fusicatenibacter | k_Bacteria;p_Firmicutes;c_Bacilli;o_Erysipelotrichales;f_Erysipelatoclostridiaceae;g_Erysipelatoclostridium | -0.653299279 | 1.64E-04 | 0.028065138 |
| k_Bacteria;p_Firmicutes;c_Bacilli;o_Erysipelotrichales;f_Erysipelotrichaceae;g_Faecalitalea | k_Bacteria;p_Firmicutes;c_Bacilli;o_Erysipelotrichales;f_Erysipelotrichaceae;g_Clostridium_innocuum | 0.642450152 | 2.28E-04 | 0.034384553 |
| k_Bacteria;p_Firmicutes;c_Clostridia;o_Peptostreptococcales_Tissierellales;f_Anaerovoracaceae;g_Family_XIII_UCG_001 | k_Bacteria;p_Firmicutes;c_Bacilli;o_Erysipelotrichales;f_Erysipelotrichaceae;g_Holdemanella | 0.611111111 | 5.51E-04 | 0.059911637 |
| k_Bacteria;p_Firmicutes;c_Clostridia;o_Clostridia_vadinBB60;f_Clostridia_vadinBB60;g_Clostridia_vadinBB60 | k_Bacteria;p_Firmicutes;c_Clostridia;o_Christensenellales;f_Christensenellaceae;g_Christensenellaceae_R_7 | 0.674590748 | 8.25E-05 | 0.019052335 |
| k_Bacteria;p_Firmicutes;c_Clostridia;o_Lachnospirales;f_Lachnospiraceae;g_Lachnospiraceae_NC2004 | k_Bacteria;p_Firmicutes;c_Clostridia;o_Christensenellales;f_Christensenellaceae;g_Christensenellaceae_R_7 | 0.671618875 | 9.11E-05 | 0.019656126 |
| k_Bacteria;p_Firmicutes;c_Clostridia;o_Lachnospirales;f_Lachnospiraceae;g_Lachnospiraceae_NK4A136 | k_Bacteria;p_Firmicutes;c_Clostridia;o_Christensenellales;f_Christensenellaceae;g_Christensenellaceae_R_7 | 0.751704569 | 4.00E-06 | 0.002548907 |
| k_Bacteria;p_Firmicutes;c_Clostridia;o_Lachnospirales;f_Lachnospiraceae;g_Lachnospiraceae_UCG_001 | k_Bacteria;p_Firmicutes;c_Clostridia;o_Christensenellales;f_Christensenellaceae;g_Christensenellaceae_R_7 | 0.821581175 | 8.38E-08 | 1.24E-04 |
| k_Bacteria;p_Firmicutes;c_Clostridia;o_Lachnospirales;f_Lachnospiraceae;g_Ruminococcus_gauvreauii | k_Bacteria;p_Firmicutes;c_Clostridia;o_Christensenellales;f_Christensenellaceae;g_Christensenellaceae_R_7 | 0.66801813 | 1.03E-04 | 0.020531352 |

| | | | | |
|---|---|---|---|---|
| k_Bacteria;p_Firmicutes;c_Clostridia;o_Monoglobales;f_Monoglobaceae;g_Monoglobus | k_Bacteria;p_Firmicutes;c_Clostridia;o_Christensenellales;f_Christensenellaceae;g_Christensenellaceae_R_7 | 0.600850164 | 7.22E-04 | 0.069186955 |
| k_Bacteria;p_Firmicutes;c_Clostridia;o_Oscillospirales;f_Eubacterium_coprostanoligenes;g_Eubacterium_coprostanoligenes | k_Bacteria;p_Firmicutes;c_Clostridia;o_Christensenellales;f_Christensenellaceae;g_Christensenellaceae_R_7 | 0.752194057 | 3.92E-06 | 0.002548907 |
| k_Bacteria;p_Firmicutes;c_Clostridia;o_Oscillospirales;f_Ruminococcaceae;g_Eubacterium_siraeum | k_Bacteria;p_Firmicutes;c_Clostridia;o_Christensenellales;f_Christensenellaceae;g_Christensenellaceae_R_7 | 0.696097918 | 3.90E-05 | 0.012862362 |
| k_Bacteria;p_Firmicutes;c_Clostridia;o_Peptostreptococcales_Tissierellales;f_Anaerovoracaceae;g_Family_XIII_AD3011 | k_Bacteria;p_Firmicutes;c_Clostridia;o_Christensenellales;f_Christensenellaceae;g_Christensenellaceae_R_7 | 0.730524104 | 1.02E-05 | 0.004528124 |
| k_Bacteria;p_Firmicutes;c_Clostridia;o_Lachnospirales;f_Lachnospiraceae;g_Lachnospiraceae_UCG_001 | k_Bacteria;p_Firmicutes;c_Clostridia;o_Clostridia_UCG_014;f_Clostridia_UCG_014;g_Clostridia_UCG_014 | 0.711249962 | 2.21E-05 | 0.008198748 |
| k_Bacteria;p_Firmicutes;c_Clostridia;o_Lachnospirales;f_Lachnospiraceae;g_Lachnospiraceae_FCS020 | k_Bacteria;p_Firmicutes;c_Clostridia;o_Clostridia_vadinBB60;f_Clostridia_vadinBB60;g_Clostridia_vadinBB60 | 0.724889217 | 1.28E-05 | 0.00544653 |
| k_Bacteria;p_Firmicutes;c_Clostridia;o_Lachnospirales;f_Lachnospiraceae;g_Lachnospiraceae_UCG_001 | k_Bacteria;p_Firmicutes;c_Clostridia;o_Clostridia_vadinBB60;f_Clostridia_vadinBB60;g_Clostridia_vadinBB60 | 0.723348345 | 1.37E-05 | 0.005536282 |
| k_Bacteria;p_Firmicutes;c_Clostridia;o_Oscillospirales;f_Ruminococcaceae;g_Eubacterium_siraeum | k_Bacteria;p_Firmicutes;c_Clostridia;o_Clostridia_vadinBB60;f_Clostridia_vadinBB60;g_Clostridia_vadinBB60 | 0.680576071 | 6.74E-05 | 0.017857232 |
| k_Bacteria;p_Firmicutes;c_Clostridia;o_Peptococcales;f_Peptococcaceae;g_Peptococcus | k_Bacteria;p_Firmicutes;c_Clostridia;o_Clostridia_vadinBB60;f_Clostridia_vadinBB60;g_Clostridia_vadinBB60 | 0.677394971 | 7.51E-05 | 0.018087092 |
| k_Bacteria;p_Firmicutes;c_Clostridia;o_Lachnospirales;f_Lachnospiraceae;g_Lachnospiraceae_UCG_001 | k_Bacteria;p_Firmicutes;c_Clostridia;o_Lachnospirales;f_Lachnospiraceae;g_Eubacterium_eligens | 0.636563857 | 2.71E-04 | 0.039557672 |
| k_Bacteria;p_Firmicutes;c_Clostridia;o_Oscillospirales;f_Ruminococcaceae;g_Ruminococcus | k_Bacteria;p_Firmicutes;c_Clostridia;o_Lachnospirales;f_Lachnospiraceae;g_Eubacterium_eligens | 0.612543267 | 5.31E-04 | 0.059911637 |
| k_Bacteria;p_Firmicutes;c_Clostridia;o_Lachnospirales;f_Lachnospiraceae;g_Fusicatenibacter | k_Bacteria;p_Firmicutes;c_Clostridia;o_Lachnospirales;f_Lachnospiraceae;g_Eubacterium_hallii | 0.686218034 | 5.54E-05 | 0.015936016 |
| k_Bacteria;p_Firmicutes;c_Clostridia;o_Lachnospirales;f_Lachnospiraceae;g_Lachnospiraceae_UCG_001 | k_Bacteria;p_Firmicutes;c_Clostridia;o_Lachnospirales;f_Lachnospiraceae;g_Eubacterium_oxidoreducens | 0.633851025 | 2.93E-04 | 0.041497623 |

| | | | | |
|---|---|---|---|---|
| k_Bacteria;p_Firmicutes;c_Clostridia;o_Peptostreptococcales_Tissierellales;f_Anaerovoracaceae;g_Family_XIII_UCG_001 | k_Bacteria;p_Firmicutes;c_Clostridia;o_Lachnospirales;f_Lachnospiraceae;g_Eubacterium_oxidoreducens | 0.644937142 | 2.11E-04 | 0.033450035 |
| k_Bacteria;p_Proteobacteria;c_Gammaproteobacteria;o_Burkholderiales;f_Oxalobacteraceae;g_Oxalobacter | k_Bacteria;p_Firmicutes;c_Clostridia;o_Lachnospirales;f_Lachnospiraceae;g_Eubacterium_oxidoreducens | 0.61358308 | 5.16E-04 | 0.059911637 |
| k_Bacteria;p_Proteobacteria;c_Gammaproteobacteria;o_Xanthomonadales;f_Xanthomonadaceae;g_Stenotrophomonas | k_Bacteria;p_Firmicutes;c_Clostridia;o_Lachnospirales;f_Lachnospiraceae;g_Eubacterium_oxidoreducens | 0.622995352 | 3.99E-04 | 0.051479976 |
| k_Bacteria;p_Firmicutes;c_Clostridia;o_Oscillospirales;f_Oscillospiraceae;g_NK4A214 | k_Bacteria;p_Firmicutes;c_Clostridia;o_Lachnospirales;f_Lachnospiraceae;g_Eubacterium_ventriosum | 0.605465334 | 6.40E-04 | 0.064106662 |
| k_Bacteria;p_Firmicutes;c_Clostridia;o_Peptostreptococcales_Tissierellales;f_Peptostreptococcaceae;g_Intestinibacter | k_Bacteria;p_Firmicutes;c_Clostridia;o_Lachnospirales;f_Lachnospiraceae;g_Eubacterium_ventriosum | 0.602718495 | 6.88E-04 | 0.067363391 |
| k_Bacteria;p_Firmicutes;c_Clostridia;o_Peptococcales;f_Peptococcaceae;g_Peptococcus | k_Bacteria;p_Firmicutes;c_Clostridia;o_Lachnospirales;f_Lachnospiraceae;g_Lachnospiraceae_FCS020 | 0.679408031 | 7.01E-05 | 0.017857232 |
| k_Bacteria;p_Firmicutes;c_Clostridia;o_Peptostreptococcales_Tissierellales;f_Anaerovoracaceae;g_Family_XIII_AD3011 | k_Bacteria;p_Firmicutes;c_Clostridia;o_Lachnospirales;f_Lachnospiraceae;g_Lachnospiraceae_NC2004 | 0.665365612 | 1.12E-04 | 0.020755384 |
| k_Bacteria;p_Firmicutes;c_Negativicutes;o_Veillonellales_Selenomonadales;f_Veillonellaceae;g_Megasphaera | k_Bacteria;p_Firmicutes;c_Clostridia;o_Lachnospirales;f_Lachnospiraceae;g_Lachnospiraceae_NC2004 | 0.681180431 | 6.60E-05 | 0.017857232 |
| k_Bacteria;p_Firmicutes;c_Clostridia;o_Lachnospirales;f_Lachnospiraceae;g_Lachnospiraceae_UCG_001 | k_Bacteria;p_Firmicutes;c_Clostridia;o_Lachnospirales;f_Lachnospiraceae;g_Lachnospiraceae_NK4A136 | 0.674283683 | 8.34E-05 | 0.019052335 |
| k_Bacteria;p_Firmicutes;c_Clostridia;o_Monoglobales;f_Monoglobaceae;g_Monoglobus | k_Bacteria;p_Firmicutes;c_Clostridia;o_Lachnospirales;f_Lachnospiraceae;g_Lachnospiraceae_NK4A136 | 0.757734131 | 3.02E-06 | 0.002242982 |
| k_Bacteria;p_Firmicutes;c_Clostridia;o_Oscillospirales;f_Eubacterium_coprostanoligenes;g_Eubacterium_coprostanoligenes | k_Bacteria;p_Firmicutes;c_Clostridia;o_Lachnospirales;f_Lachnospiraceae;g_Lachnospiraceae_NK4A136 | 0.720391329 | 1.54E-05 | 0.005967725 |
| k_Bacteria;p_Firmicutes;c_Clostridia;o_Peptostreptococcales_Tissierellales;f_Anaerovoracaceae;g_Family_XIII_AD3011 | k_Bacteria;p_Firmicutes;c_Clostridia;o_Lachnospirales;f_Lachnospiraceae;g_Lachnospiraceae_NK4A136 | 0.667378999 | 1.05E-04 | 0.020531352 |
| k_Bacteria;p_Firmicutes;c_Clostridia;o_Oscillospirales;f_Eubacterium_coprostanoligenes;g_Eubacterium_coprostanoligenes | k_Bacteria;p_Firmicutes;c_Clostridia;o_Lachnospirales;f_Lachnospiraceae;g_Lachnospiraceae_UCG_001 | 0.707271896 | 2.57E-05 | 0.009167815 |

| | | | | |
|---|---|---|---|---|
| k_Bacteria;p_Firmicutes;c_Clostridia;o_Oscillospirales;f_Ruminococcaceae;g_Eubacterium_siraeum | k_Bacteria;p_Firmicutes;c_Clostridia;o_Lachnospirales;f_Lachnospiraceae;g_Lachnospiraceae_UCG_001 | 0.739005235 | 7.07E-06 | 0.003697584 |
| k_Bacteria;p_Firmicutes;c_Clostridia;o_Peptostreptococcales_Tissierellales;f_Anaerovoracaceae;g_Family_XIII_AD3011 | k_Bacteria;p_Firmicutes;c_Clostridia;o_Lachnospirales;f_Lachnospiraceae;g_Lachnospiraceae_UCG_001 | 0.654186503 | 1.59E-04 | 0.027839096 |
| k_Bacteria;p_Firmicutes;c_Clostridia;o_Peptostreptococcales_Tissierellales;f_Anaerovoracaceae;g_Family_XIII_UCG_001 | k_Bacteria;p_Firmicutes;c_Clostridia;o_Lachnospirales;f_Lachnospiraceae;g_Lachnospiraceae_UCG_001 | 0.60932352 | 5.78E-04 | 0.061340644 |
| k_Bacteria;p_Proteobacteria;c_Gammaproteobacteria;o_Burkholderiales;f_Oxalobacteraceae;g_Oxalobacter | k_Bacteria;p_Firmicutes;c_Clostridia;o_Lachnospirales;f_Lachnospiraceae;g_Lachnospiraceae_UCG_001 | 0.644525913 | 2.14E-04 | 0.033450035 |
| k_Bacteria;p_Firmicutes;c_Clostridia;o_Oscillospirales;f_Ruminococcaceae;g_Faecalibacterium | k_Bacteria;p_Firmicutes;c_Clostridia;o_Lachnospirales;f_Lachnospiraceae;g_Lachnospiraceae_UCG_004 | 0.609853589 | 5.70E-04 | 0.061210159 |
| k_Bacteria;p_Proteobacteria;c_Gammaproteobacteria;o_Enterobacterales;f_Enterobacteriaceae;g_Escherichia_Shigella | k_Bacteria;p_Firmicutes;c_Clostridia;o_Lachnospirales;f_Lachnospiraceae;g_Roseburia | -0.612716131 | 5.28E-04 | 0.059911637 |
| k_Bacteria;p_Firmicutes;c_Clostridia;o_Oscillospirales;f_Ruminococcaceae;g_Eubacterium_siraeum | k_Bacteria;p_Firmicutes;c_Clostridia;o_Lachnospirales;f_Lachnospiraceae;g_Ruminococcus_gauvreauii | 0.759512427 | 2.78E-06 | 0.002242982 |
| k_Bacteria;p_Firmicutes;c_Clostridia;o_Peptostreptococcales_Tissierellales;f_Anaerovoracaceae;g_Family_XIII_AD3011 | k_Bacteria;p_Firmicutes;c_Clostridia;o_Lachnospirales;f_Lachnospiraceae;g_Ruminococcus_gauvreauii | 0.607363576 | 6.09E-04 | 0.061674054 |
| k_Bacteria;p_Firmicutes;c_Clostridia;o_Oscillospirales;f_Oscillospiraceae;g_NK4A214 | k_Bacteria;p_Firmicutes;c_Clostridia;o_Oscillospirales;f_Eubacterium_coprostanoligenes;g_Eubacterium_coprostanoligenes | 0.764805329 | 2.15E-06 | 0.00191357 |
| k_Bacteria;p_Firmicutes;c_Clostridia;o_Oscillospirales;f_Ruminococcaceae;g_Ruminococcus | k_Bacteria;p_Firmicutes;c_Clostridia;o_Oscillospirales;f_Eubacterium_coprostanoligenes;g_Eubacterium_coprostanoligenes | 0.692635903 | 4.42E-05 | 0.013570443 |
| k_Bacteria;p_Firmicutes;c_Clostridia;o_Peptostreptococcales_Tissierellales;f_Anaerovoracaceae;g_Family_XIII_AD3011 | k_Bacteria;p_Firmicutes;c_Clostridia;o_Oscillospirales;f_Eubacterium_coprostanoligenes;g_Eubacterium_coprostanoligenes | 0.736321143 | 7.94E-06 | 0.003726189 |
| k_Bacteria;p_Firmicutes;c_Clostridia;o_Oscillospirales;f_Ruminococcaceae;g_Negativibacillus | k_Bacteria;p_Firmicutes;c_Clostridia;o_Oscillospirales;f_Oscillospiraceae;g_NK4A214 | 0.737752684 | 7.47E-06 | 0.003697584 |

| | | | | |
|---|---|---|---|---|
| k_Bacteria;p_Firmicutes;c_Clostridia;o_Peptostreptococcales_Tissierellales;f_Anaerovoracaceae;g_Family_XIII_UCG_001 | k_Bacteria;p_Firmicutes;c_Clostridia;o_Oscillospirales;f_Oscillospiraceae;g_NK4A214 | 0.624237033 | 3.85E-04 | 0.0512116 |
| k_Bacteria;p_Firmicutes;c_Clostridia;o_Peptococcales;f_Peptococcaceae;g_Peptococcus | k_Bacteria;p_Firmicutes;c_Clostridia;o_Oscillospirales;f_Ruminococcaceae;g_Eubacterium_siraeum | 0.650218649 | 1.80E-04 | 0.029173582 |
| k_Bacteria;p_Firmicutes;c_Clostridia;o_Peptostreptococcales_Tissierellales;f_Anaerovoracaceae;g_Family_XIII_AD3011 | k_Bacteria;p_Firmicutes;c_Clostridia;o_Oscillospirales;f_Ruminococcaceae;g_Eubacterium_siraeum | 0.651674069 | 1.72E-04 | 0.028415762 |
| k_Bacteria;p_Proteobacteria;c_Gammaproteobacteria;o_Burkholderiales;f_Oxalobacteraceae;g_Oxalobacter | k_Bacteria;p_Firmicutes;c_Clostridia;o_Oscillospirales;f_Ruminococcaceae;g_Eubacterium_siraeum | 0.806951431 | 2.14E-07 | 2.38E-04 |
| k_Bacteria;p_Firmicutes;c_Clostridia;o_Peptostreptococcales_Tissierellales;f_Anaerovoracaceae;g_Family_XIII_UCG_001 | k_Bacteria;p_Firmicutes;c_Clostridia;o_Oscillospirales;f_Ruminococcaceae;g_Negativibacillus | 0.811521928 | 1.61E-07 | 2.05E-04 |
| k_Bacteria;p_Proteobacteria;c_Gammaproteobacteria;o_Burkholderiales;f_Oxalobacteraceae;g_Oxalobacter | k_Bacteria;p_Firmicutes;c_Clostridia;o_Peptococcales;f_Peptococcaceae;g_Peptococcus | 0.765488112 | 2.08E-06 | 0.00191357 |
| k_Bacteria;p_Proteobacteria;c_Gammaproteobacteria;o_Burkholderiales;f_Oxalobacteraceae;g_Oxalobacter | k_Bacteria;p_Firmicutes;c_Clostridia;o_Peptostreptococcales_Tissierellales;f_Anaerovoracaceae;g_Family_XIII_AD3011 | 0.623607673 | 3.92E-04 | 0.051353204 |
| k_Bacteria;p_Proteobacteria;c_Gammaproteobacteria;o_Burkholderiales;f_Oxalobacteraceae;g_Oxalobacter | k_Bacteria;p_Firmicutes;c_Clostridia;o_Peptostreptococcales_Tissierellales;f_Anaerovoracaceae;g_Family_XIII_UCG_001 | 0.666350336 | 1.08E-04 | 0.020531352 |

**Table S2** The results of Spearman abundance correlation analysis of TB2MT group at the genus level. Only significant correlations with p≤0.10 after false discovery rate correction and |r| ≥ 0.6 are displayed.

| **genus_a** | **genus_b** | **r** | **p value** | **p(FDR)** |
|---|---|---|---|---|
| k_Bacteria;p_Proteobacteria;c_Gammaproteobacteria;o_Burkholderiales;f_Alcaligenaceae;g_Achromobacter | k_Fungi;p_Basidiomycota;c_Tremellomycetes;o_Tremellales;f_Tremellaceae;g_Cryptococcus | 0.719622917 | 1.08E-05 | 0.012073 |
| k_Bacteria;p_Bacteroidota;c_Bacteroidia;o_Bacteroidales;f_Prevotellace | k_Bacteria;p_Bacteroidota;c_Bacteroidia;o_Bacteroidales;f_Bacteroida | - | 1.04E-05 | 0.012073 |

| | | | | |
|---|---|---|---|---|
| ae;g_Prevotella | ceae;g_Bacteroides | 0.720523729 | | |
| k_Bacteria;p_Bacteroidota;c_Bacteroidia;o_Bacteroidales;f_Barnesiellaceae;g_Coprobacter | k_Bacteria;p_Bacteroidota;c_Bacteroidia;o_Bacteroidales;f_Barnesiellaceae;g_Barnesiella | 0.719622917 | 1.08E-05 | 0.012073 |
| k_Bacteria;p_Firmicutes;c_Clostridia;o_Peptostreptococcales_Tissierellales;f_Peptostreptococcaceae;g_Terrisporobacter | k_Bacteria;p_Bacteroidota;c_Bacteroidia;o_Bacteroidales;f_Barnesiellaceae;g_Barnesiella | 0.719622917 | 1.08E-05 | 0.012073 |
| k_Bacteria;p_Firmicutes;c_Clostridia;o_Peptostreptococcales_Tissierellales;f_Peptostreptococcaceae;g_Terrisporobacter | k_Bacteria;p_Bacteroidota;c_Bacteroidia;o_Bacteroidales;f_Barnesiellaceae;g_Coprobacter | 1 | 0 | 0 |
| k_Bacteria;p_Firmicutes;c_Clostridia;o_Peptostreptococcales_Tissierellales;f_Anaerovoracaceae;g_Family_XIII_AD3011 | k_Bacteria;p_Bacteroidota;c_Bacteroidia;o_Bacteroidales;f_Marinifilaceae;g_Butyricimonas | 0.685458082 | 4.07E-05 | 0.036303 |
| k_Bacteria;p_Firmicutes;c_Clostridia;o_Lachnospirales;f_Lachnospiraceae;g_Lachnospiraceae_ND3007 | k_Bacteria;p_Bacteroidota;c_Bacteroidia;o_Bacteroidales;f_Marinifilaceae;g_Odoribacter | 0.691276712 | 3.29E-05 | 0.032601 |
| k_Bacteria;p_Firmicutes;c_Clostridia;o_Lachnospirales;f_Lachnospiraceae;g_Lachnospiraceae_ND3007 | k_Bacteria;p_Bacteroidota;c_Bacteroidia;o_Bacteroidales;f_Rikenellaceae;g_Alistipes | 0.667355341 | 7.67E-05 | 0.049174 |
| k_Bacteria;p_Firmicutes;c_Clostridia;o_Oscillospirales;f_Ruminococcaceae;g_Subdoligranulum | k_Bacteria;p_Firmicutes;c_Clostridia;o_Lachnospirales;f_Lachnospiraceae;g_Agathobacter | 0.667150012 | 7.73E-05 | 0.049174 |
| k_Bacteria;p_Firmicutes;c_Clostridia;o_Lachnospirales;f_Lachnospirace | k_Bacteria;p_Firmicutes;c_Clostridia;o_Lachnospirales;f_Lachnospira | 0.74232499 | 4.03E-06 | 0.011958 |

| | | | | |
|---|---|---|---|---|
| ae;g_Eubacterium_hallii | ceae;g_Dorea | | | |
| k_Bacteria;p_Firmicutes;c_Clostridia;o_Peptostreptococcales_Tissierellales;f_Anaerovoracaceae;g_Family_XIII_UCG_001 | k_Bacteria;p_Firmicutes;c_Clostridia;o_Lachnospirales;f_Lachnospiraceae;g_Lachnospiraceae_FCS020 | 0.719622917 | 1.08E-05 | 0.012073 |
| k_Bacteria;p_Firmicutes;c_Clostridia;o_Monoglobales;f_Monoglobaceae;g_Monoglobus | k_Bacteria;p_Firmicutes;c_Clostridia;o_Lachnospirales;f_Lachnospiraceae;g_Lachnospiraceae_UCG_001 | 0.661015191 | 9.48E-05 | 0.056327 |
| k_Bacteria;p_Proteobacteria;c_Gammaproteobacteria;o_Burkholderiales;f_Oxalobacteraceae;g_Oxalobacter | k_Bacteria;p_Firmicutes;c_Clostridia;o_Oscillospirales;f_Oscillospiraceae;g_NK4A214 | 0.66822128 | 7.45E-05 | 0.049174 |
| k_Bacteria;p_Proteobacteria;c_Gammaproteobacteria;o_Pasteurellales;f_Pasteurellaceae;g_Haemophilus | k_Bacteria;p_Firmicutes;c_Clostridia;o_Oscillospirales;f_Oscillospiraceae;g_NK4A214 | 0.801430439 | 1.75E-07 | 7.80E-04 |
| k_Bacteria;p_Proteobacteria;c_Gammaproteobacteria;o_Burkholderiales;f_Oxalobacteraceae;g_Oxalobacter | k_Bacteria;p_Firmicutes;c_Clostridia;o_Peptostreptococcales_Tissierellales;f_Anaerovoracaceae;g_Family_XIII_UCG_001 | 0.66822128 | 7.45E-05 | 0.049174 |

**Table S3** The results of Spearman abundance correlation analysis of TB6MT group at the genus level. Only significant correlations with p≤0.10 after false discovery rate correction and |r| ≥ 0.6 are displayed.

| genus_a | genus_b | r | p value | p(FDR) |
|---|---|---|---|---|
| k_Fungi;p_Ascomycota;c_Eurotiomycetes;o_Chaetothyriales;f_Herpotrichiellaceae;g_Phialophora | k_Fungi;p_Ascomycota;c_Dothideomycetes;o_Capnodiales;f_Cladosporiaceae;g_Cladosporium | 0.763456622 | 1.43E-04 | 0.066915869 |

| | | | | |
|---|---|---|---|---|
| k_Fungi;p_Basidiomycota;c_Microbotryomycetes;o_Sporidiobolales;f_Sporidiobolaceae;g_Rhodotorula | k_Fungi;p_Ascomycota;c_Dothideomycetes;o_Capnodiales;f_Cladosporiaceae;g_Cladosporium | 0.764478479 | 1.38E-04 | 0.066915869 |
| k_Bacteria;p_Firmicutes;c_Clostridia;o_Lachnospirales;f_Lachnospiraceae;g_Eubacterium_oxidoreducens | k_Fungi;p_Ascomycota;c_Dothideomycetes;o_Pleosporales;f_Sporormiaceae;g_Preussia | 0.764060308 | 1.40E-04 | 0.066915869 |
| k_Fungi;p_Basidiomycota;c_Wallemiomycetes;o_Wallemiales;f_Wallemiaceae;g_Wallemia | k_Fungi;p_Ascomycota;c_Eurotiomycetes;o_Eurotiales;f_Aspergillaceae;g_Aspergillus | 0.747108382 | 2.37E-04 | 0.083528931 |
| k_Fungi;p_Ascomycota;c_Sordariomycetes;o_Sordariales;f_Chaetomiaceae;g_Chaetomium | k_Fungi;p_Ascomycota;c_Saccharomycetes;o_Saccharomycetales;f_Debaryomycetaceae;g_Meyerozyma | 0.80468873 | 3.26E-05 | 0.02107149 |
| k_Bacteria;p_Firmicutes;c_Clostridia;o_Lachnospirales;f_Lachnospiraceae;g_Lachnospiraceae_ND3007 | k_Fungi;p_Ascomycota;c_Saccharomycetes;o_Saccharomycetales;f_Saccharomycetaceae;g_Saccharomyces | -0.728914386 | 4.00E-04 | 0.091983371 |
| k_Bacteria;p_Firmicutes;c_Bacilli;o_Lactobacillales;f_Streptococcaceae;g_Streptococcus | k_Fungi;p_Ascomycota;c_Sordariomycetes;o_Hypocreales;f_Nectriaceae;g_Gibberella | -0.76045555 | 1.57E-04 | 0.067678414 |
| k_Bacteria;p_Firmicutes;c_Clostridia;o_Clostridiales;f_Clostridiaceae;g_Clostridium_sensu_stricto_1 | k_Fungi;p_Basidiomycota;c_Agaricomycetes;o_Agaricales;f_Pleurotaceae;g_Pleurotus | 0.756534362 | 1.78E-04 | 7.25E-02 |
| k_Bacteria;p_Firmicutes;c_Negativicutes;o_Veillonellales_Selenomonadales;f_Veillonellaceae;g_Dialister | k_Fungi;p_Basidiomycota;c_Agaricomycetes;o_Agaricales;f_Pleurotaceae;g_Pleurotus | 0.730548891 | 3.82E-04 | 0.091983371 |
| k_Bacteria;p_Firmicutes;c_Bacilli;o_Erysipelotrichales;f_Erysipelotrich | k_Fungi;p_Basidiomycota;c_Tremellomycetes;o_Filobasidiales;f_Filo | 1 | 6.08E- | 9.4224E |

| | | | | |
|---|---|---|---|---|
| aceae;g_Holdemanella | basidiaceae;g_Naganishia | | 132 | -129 |
| k_Bacteria;p_Firmicutes;c_Clostridia;o_Oscillospirales;f_Oscillospiraceae;g_NK4A214 | k_Fungi;p_Mucoromycota;c_Mucoromycetes;o_Mucorales;f_Mucoraceae;g_Mucor | 0.726483157 | 4.27E-04 | 0.091983371 |
| k_Bacteria;p_Bacteroidota;c_Bacteroidia;o_Bacteroidales;f_Barnesiellaceae;g_Coprobacter | k_Bacteria;p_Bacteroidota;c_Bacteroidia;o_Bacteroidales;f_Barnesiellaceae;g_Barnesiella | 0.726483157 | 4.27E-04 | 0.091983371 |
| k_Bacteria;p_Firmicutes;c_Clostridia;o_Lachnospirales;f_Lachnospiraceae;g_Eubacterium_eligens | k_Bacteria;p_Bacteroidota;c_Bacteroidia;o_Bacteroidales;f_Marinifilaceae;g_Odoribacter | 0.834073745 | 9.0765E-06 | 0.006394806 |
| k_Bacteria;p_Firmicutes;c_Clostridia;o_Lachnospirales;f_Lachnospiraceae;g_Ruminococcus_gauvreauii | k_Bacteria;p_Bacteroidota;c_Bacteroidia;o_Bacteroidales;f_Marinifilaceae;g_Odoribacter | 0.839254327 | 7.06E-06 | 5.80E-03 |
| k_Bacteria;p_Firmicutes;c_Clostridia;o_Peptostreptococcales_Tissierellales;f_Anaerovoracaceae;g_Family_XIII_AD3011 | k_Bacteria;p_Bacteroidota;c_Bacteroidia;o_Bacteroidales;f_Marinifilaceae;g_Odoribacter | 0.839254327 | 7.06E-06 | 5.80E-03 |
| k_Bacteria;p_Proteobacteria;c_Gammaproteobacteria;o_Xanthomonadales;f_Xanthomonadaceae;g_Stenotrophomonas | k_Bacteria;p_Bacteroidota;c_Bacteroidia;o_Bacteroidales;f_Muribaculaceae;g_Muribaculaceae | 0.76260079 | 1.47E-04 | 6.69E-02 |
| k_Bacteria;p_Firmicutes;c_Clostridia;o_Monoglobales;f_Monoglobaceae;g_Monoglobus | k_Bacteria;p_Desulfobacterota;c_Desulfovibrionia;o_Desulfovibrionales;f_Desulfovibrionaceae;g_Bilophila | 0.763882021 | 1.41E-04 | 6.69E-02 |
| k_Bacteria;p_Firmicutes;c_Clostridia;o_Oscillospirales;f_Ruminococcaceae;g_Candidatus_Soleaferrea | k_Bacteria;p_Firmicutes;c_Bacilli;o_Lactobacillales;f_Enterococcaceae;g_Enterococcus | 0.726483157 | 4.27E-04 | 0.091983371 |

| | | | | |
|---|---|---|---|---|
| k_Bacteria;p_Firmicutes;c_Negativicutes;o_Acidaminococcales;f_Acidaminococcaceae;g_Phascolarctobacterium | k_Bacteria;p_Firmicutes;c_Bacilli;o_Lactobacillales;f_Streptococcaceae;g_Streptococcus | -0.740299011 | 2.90E-04 | 9.20E-02 |
| k_Bacteria;p_Firmicutes;c_Clostridia;o_Lachnospirales;f_Lachnospiraceae;g_Lachnospiraceae_FCS020 | k_Bacteria;p_Firmicutes;c_Clostridia;o_Lachnospirales;f_Lachnospiraceae;g_Eubacterium_eligens | 0.726483157 | 4.27E-04 | 0.091983371 |
| k_Bacteria;p_Firmicutes;c_Clostridia;o_Lachnospirales;f_Lachnospiraceae;g_Lachnospiraceae_NK4A136 | k_Bacteria;p_Firmicutes;c_Clostridia;o_Lachnospirales;f_Lachnospiraceae;g_Eubacterium_eligens | 0.726483157 | 0.000427278 | 0.091983371 |
| k_Bacteria;p_Firmicutes;c_Clostridia;o_Lachnospirales;f_Lachnospiraceae;g_Ruminococcus_gauvreauii | k_Bacteria;p_Firmicutes;c_Clostridia;o_Lachnospirales;f_Lachnospiraceae;g_Eubacterium_eligens | 0.99382716 | 1.12E-17 | 1.23996E-14 |
| k_Bacteria;p_Firmicutes;c_Clostridia;o_Monoglobales;f_Monoglobaceae;g_Monoglobus | k_Bacteria;p_Firmicutes;c_Clostridia;o_Lachnospirales;f_Lachnospiraceae;g_Eubacterium_eligens | 0.742795681 | 0.00026938 | 0.090769438 |
| k_Bacteria;p_Firmicutes;c_Clostridia;o_Peptostreptococcales_Tissierellales;f_Anaerovoracaceae;g_Family_XIII_AD3011 | k_Bacteria;p_Firmicutes;c_Clostridia;o_Lachnospirales;f_Lachnospiraceae;g_Eubacterium_eligens | 0.99382716 | 1.11996E-17 | 1.23996E-14 |
| k_Bacteria;p_Firmicutes;c_Clostridia;o_Peptostreptococcales_Tissierellales;f_Anaerovoracaceae;g_Family_XIII_UCG_001 | k_Bacteria;p_Firmicutes;c_Clostridia;o_Lachnospirales;f_Lachnospiraceae;g_Eubacterium_eligens | 0.726483157 | 0.000427278 | 0.091983371 |
| k_Bacteria;p_Firmicutes;c_Clostridia;o_Oscillospirales;f_Eubacterium_coprostanoligenes;g_Eubacterium_coprostanoligenes | k_Bacteria;p_Firmicutes;c_Clostridia;o_Lachnospirales;f_Lachnospiraceae;g_Eubacterium_oxidoreducens | 0.73297674 | 0.000356982 | 0.091983371 |
| k_Bacteria;p_Firmicutes;c_Clostridia;o_Lachnospirales;f_Lachnospirace | k_Bacteria;p_Firmicutes;c_Clostridia;o_Lachnospirales;f_Lachnospira | 1 | 6.079E- | 9.4224E |

| | | | | |
|---|---|---|---|---|
| ae;g_Lachnospiraceae_NK4A136 | ceae;g_Lachnospiraceae_FCS020 | | 132 | -129 |
| k_Bacteria;p_Firmicutes;c_Clostridia;o_Peptostreptococcales_Tissierellales;f_Anaerovoracaceae;g_Family_XIII_UCG_001 | k_Bacteria;p_Firmicutes;c_Clostridia;o_Lachnospirales;f_Lachnospiraceae;g_Lachnospiraceae_FCS020 | 1 | 6.08E-132 | 9.4224E-129 |
| k_Bacteria;p_Firmicutes;c_Clostridia;o_Lachnospirales;f_Lachnospiraceae;g_Roseburia | k_Bacteria;p_Firmicutes;c_Clostridia;o_Lachnospirales;f_Lachnospiraceae;g_Lachnospiraceae_ND3007 | 0.838076781 | 7.48207E-06 | 0.005798605 |
| k_Bacteria;p_Firmicutes;c_Clostridia;o_Peptostreptococcales_Tissierellales;f_Anaerovoracaceae;g_Family_XIII_UCG_001 | k_Bacteria;p_Firmicutes;c_Clostridia;o_Lachnospirales;f_Lachnospiraceae;g_Lachnospiraceae_NK4A136 | 1 | 6.079E-132 | 9.4224E-129 |
| k_Bacteria;p_Firmicutes;c_Clostridia;o_Lachnospirales;f_Lachnospiraceae;g_Ruminococcus_gauvreauii | k_Bacteria;p_Firmicutes;c_Clostridia;o_Lachnospirales;f_Lachnospiraceae;g_Lachnospiraceae_UCG_001 | 0.726483157 | 0.000427278 | 0.091983371 |
| k_Bacteria;p_Firmicutes;c_Clostridia;o_Peptostreptococcales_Tissierellales;f_Anaerovoracaceae;g_Family_XIII_AD3011 | k_Bacteria;p_Firmicutes;c_Clostridia;o_Lachnospirales;f_Lachnospiraceae;g_Lachnospiraceae_UCG_001 | 0.726483157 | 0.000427278 | 0.091983371 |
| k_Bacteria;p_Firmicutes;c_Clostridia;o_Monoglobales;f_Monoglobaceae;g_Monoglobus | k_Bacteria;p_Firmicutes;c_Clostridia;o_Lachnospirales;f_Lachnospiraceae;g_Ruminococcus_gauvreauii | 0.747409319 | 0.000234991 | 0.083528931 |
| k_Bacteria;p_Firmicutes;c_Clostridia;o_Peptostreptococcales_Tissierellales;f_Anaerovoracaceae;g_Family_XIII_AD3011 | k_Bacteria;p_Firmicutes;c_Clostridia;o_Lachnospirales;f_Lachnospiraceae;g_Ruminococcus_gauvreauii | 1 | 0 | 0 |
| k_Bacteria;p_Firmicutes;c_Clostridia;o_Peptostreptococcales_Tissierellales;f_Anaerovoracaceae;g_Family_XIII_AD3011 | k_Bacteria;p_Firmicutes;c_Clostridia;o_Monoglobales;f_Monoglobaceae;g_Monoglobus | 0.747409319 | 0.000234991 | 0.083528931 |

| | | | | |
|---|---|---|---|---|
| k_Bacteria;p_Firmicutes;c_Negativicutes;o_Veillonellales_Selenomonadales;f_Veillonellaceae;g_Veillonella | k_Bacteria;p_Firmicutes;c_Clostridia;o_Oscillospirales;f_Butyricicoccaceae;g_Butyricicoccus | -0.739040883 | 3.00E-04 | 0.091983371 |

**Table S4** The results of Spearman abundance correlation analysis of HS group at the genus level. Only significant correlations with $p \leq 0.10$ after false discovery rate correction and $|r| \geq 0.6$ are displayed.

| **genus_a** | **genus_b** | **r** | **p value** | **p(FDR)** |
|---|---|---|---|---|
| k_Fungi;p_Ascomycota;c_Eurotiomycetes;o_Chaetothyriales;f_Trichomeriaceae;g_Knufia | k_Fungi;p_Ascomycota;c_Dothideomycetes;o_Dothideales;f_Aureobasidiaceae;g_Aureobasidium | 0.648783657 | 0.001089453 | 0.091586029 |
| k_Fungi;p_Ascomycota;c_Saccharomycetes;o_Saccharomycetales;f_Phaffomycetaceae;g_Wickerhamomyces | k_Fungi;p_Ascomycota;c_Dothideomycetes;o_Dothideales;f_Aureobasidiaceae;g_Aureobasidium | 0.691396509 | 3.66E-04 | 0.05343121 |
| k_Bacteria;p_Bacteroidota;c_Bacteroidia;o_Bacteroidales;f_Prevotellaceae;g_Paraprevotella | k_Fungi;p_Ascomycota;c_Eurotiomycetes;o_Chaetothyriales;f_Herpotrichiellaceae;g_Exophiala | -0.717948398 | 1.68E-04 | 0.03659378 |
| k_Bacteria;p_Firmicutes;c_Clostridia;o_Oscillospirales;f_Oscillospiraceae;g_NK4A214 | k_Fungi;p_Ascomycota;c_Eurotiomycetes;o_Chaetothyriales;f_Herpotrichiellaceae;g_Exophiala | -0.699003856 | 2.95E-04 | 0.047845589 |
| k_Bacteria;p_Firmicutes;c_Bacilli;o_Erysipelotrichales;f_Erysipelotrichaceae;g_Holdemanella | k_Fungi;p_Ascomycota;c_Eurotiomycetes;o_Chaetothyriales;f_Herpotrichiellaceae;g_Phialophora | -0.65589389 | 9.19E-04 | 0.08186002 |
| k_Fungi;p_Ascomycota;c_Sordariomycetes;o_Hypocreales;f_Hypocreac | k_Fungi;p_Ascomycota;c_Eurotiomycetes;o_Eurotiales;f_Trichocoma | 0.659257012 | 8.46E- | 0.081077 |

| | | | | |
|---|---|---|---|---|
| eae;g_Trichoderma | ceae;g_Talaromyces | | 04 | 183 |
| k_Bacteria;p_Fusobacteriota;c_Fusobacteriia;o_Fusobacteriales;f_Fusobacteriaceae;g_Fusobacterium | k_Fungi;p_Ascomycota;c_Saccharomycetes;o_Saccharomycetales;f_Trichomonascaceae;g_Trichomonascus | 0.767456359 | 3.07E-05 | 0.016991198 |
| k_Bacteria;p_Proteobacteria;c_Gammaproteobacteria;o_Pseudomonadales;f_Moraxellaceae;g_Acinetobacter | k_Fungi;p_Ascomycota;c_Saccharomycetes;o_Saccharomycetales;f_Trichomonascaceae;g_Trichomonascus | 0.667705736 | 6.85E-04 | 0.071736373 |
| k_Bacteria;p_Actinobacteriota;c_Coriobacteriia;o_Coriobacteriales;f_Eggerthellaceae;g_Senegalimassilia | k_Fungi;p_Mucoromycota;c_Mucoromycetes;o_Mucorales;f_Mucoraceae;g_Mucor | 0.642860512 | 0.001251718 | 0.097710696 |
| k_Bacteria;p_Firmicutes;c_Negativicutes;o_Veillonellales_Selenomonadales;f_Selenomonadaceae;g_Megamonas | k_Bacteria;p_Actinobacteriota;c_Actinobacteria;o_Bifidobacteriales;f_Bifidobacteriaceae;g_Bifidobacterium | -0.743660647 | 7.29E-05 | 0.024755225 |
| k_Bacteria;p_Firmicutes;c_Clostridia;o_Lachnospirales;f_Lachnospiraceae;g_Ruminococcus_gnavus | k_Bacteria;p_Actinobacteriota;c_Actinobacteria;o_Micrococcales;f_Micrococcaceae;g_Rothia | 0.66672294 | 7.03E-04 | 0.071736373 |
| k_Bacteria;p_Actinobacteriota;c_Coriobacteriia;o_Coriobacteriales;f_Eggerthellaceae;g_Eggerthella | k_Bacteria;p_Actinobacteriota;c_Coriobacteriia;o_Coriobacteriales;f_Coriobacteriaceae;g_Collinsella | -0.64234346 | 0.00126681 | 0.097710696 |
| k_Bacteria;p_Firmicutes;c_Clostridia;o_Lachnospirales;f_Lachnospiraceae;g_Eubacterium_oxidoreducens | k_Bacteria;p_Actinobacteriota;c_Coriobacteriia;o_Coriobacteriales;f_Coriobacteriaceae;g_Collinsella | 0.771359859 | 2.63E-05 | 0.016769859 |
| k_Bacteria;p_Firmicutes;c_Clostridia;o_Oscillospirales;f_Oscillospiraceae;g_Flavonifractor | k_Bacteria;p_Actinobacteriota;c_Coriobacteriia;o_Coriobacteriales;f_Eggerthellaceae;g_Eggerthella | 0.782455037 | 1.68E-05 | 0.013720782 |

| | | | | |
|---|---|---|---|---|
| k_Bacteria;p_Firmicutes;c_Clostridia;o_Clostridia_UCG_014;f_Clostridia_UCG_014;g_Clostridia_UCG_014 | k_Bacteria;p_Actinobacteriota;c_Coriobacteriia;o_Coriobacteriales;f_Eggerthellaceae;g_Senegalimassilia | 0.64216818 | 0.001271961 | 0.097710696 |
| k_Bacteria;p_Proteobacteria;c_Gammaproteobacteria;o_Burkholderiales;f_Sutterellaceae;g_Parasutterella | k_Bacteria;p_Bacteroidota;c_Bacteroidia;o_Bacteroidales;f_Bacteroidaceae;g_Bacteroides | 0.694173294 | 3.39E-04 | 0.051798987 |
| k_Bacteria;p_Bacteroidota;c_Bacteroidia;o_Bacteroidales;f_Rikenellaceae;g_Alistipes | k_Bacteria;p_Bacteroidota;c_Bacteroidia;o_Bacteroidales;f_Barnesiellaceae;g_Barnesiella | 0.645193551 | 0.00118551 | 0.096037059 |
| k_Bacteria;p_Firmicutes;c_Clostridia;o_Lachnospirales;f_Lachnospiraceae;g_Coprococcus | k_Bacteria;p_Bacteroidota;c_Bacteroidia;o_Bacteroidales;f_Barnesiellaceae;g_Barnesiella | 0.704680164 | 2.51E-04 | 0.043799119 |
| k_Bacteria;p_Bacteroidota;c_Bacteroidia;o_Bacteroidales;f_Rikenellaceae;g_Alistipes | k_Bacteria;p_Bacteroidota;c_Bacteroidia;o_Bacteroidales;f_Marinifilaceae;g_Butyricimonas | 0.728342411 | 1.21E-04 | 0.0294995 |
| k_Bacteria;p_Firmicutes;c_Clostridia;o_Christensenellales;f_Christensenellaceae;g_Christensenellaceae_R_7 | k_Bacteria;p_Bacteroidota;c_Bacteroidia;o_Bacteroidales;f_Marinifilaceae;g_Butyricimonas | 0.766004534 | 3.24E-05 | 0.016991198 |
| k_Bacteria;p_Firmicutes;c_Clostridia;o_Lachnospirales;f_Lachnospiraceae;g_Coprococcus | k_Bacteria;p_Bacteroidota;c_Bacteroidia;o_Bacteroidales;f_Marinifilaceae;g_Butyricimonas | 0.745072019 | 6.94E-05 | 0.024755225 |
| k_Bacteria;p_Firmicutes;c_Clostridia;o_Lachnospirales;f_Lachnospiraceae;g_Lachnospiraceae_UCG_010 | k_Bacteria;p_Bacteroidota;c_Bacteroidia;o_Bacteroidales;f_Marinifilaceae;g_Butyricimonas | 0.682091675 | 4.71E-04 | 0.059983267 |
| k_Bacteria;p_Firmicutes;c_Clostridia;o_Monoglobales;f_Monoglobacea | k_Bacteria;p_Bacteroidota;c_Bacteroidia;o_Bacteroidales;f_Marinifila | 0.717068133 | 1.73E- | 0.036703 |

| | | | | |
|---|---|---|---|---|
| e;g_Monoglobus | ceae;g_Butyricimonas | | 04 | 047 |
| k_Bacteria;p_Firmicutes;c_Clostridia;o_Oscillospirales;f_Oscillospiraceae;g_NK4A214 | k_Bacteria;p_Bacteroidota;c_Bacteroidia;o_Bacteroidales;f_Marinifilaceae;g_Butyricimonas | 0.745151515 | 6.92E-05 | 0.024755225 |
| k_Bacteria;p_Firmicutes;c_Clostridia;o_Peptostreptococcales_Tissierellales;f_Anaerovoracaceae;g_Family_XIII_AD3011 | k_Bacteria;p_Bacteroidota;c_Bacteroidia;o_Bacteroidales;f_Marinifilaceae;g_Butyricimonas | 0.886715433 | 3.91E-08 | 3.48E-04 |
| k_Bacteria;p_Firmicutes;c_Clostridia;o_Clostridia_UCG_014;f_Clostridia_UCG_014;g_Clostridia_UCG_014 | k_Bacteria;p_Bacteroidota;c_Bacteroidia;o_Bacteroidales;f_Marinifilaceae;g_Odoribacter | 0.651944221 | 0.001010455 | 0.087921125 |
| k_Bacteria;p_Firmicutes;c_Clostridia;o_Lachnospirales;f_Lachnospiraceae;g_Coprococcus | k_Bacteria;p_Bacteroidota;c_Bacteroidia;o_Bacteroidales;f_Marinifilaceae;g_Odoribacter | 0.741586008 | 7.82E-05 | 0.024755225 |
| k_Bacteria;p_Firmicutes;c_Clostridia;o_Oscillospirales;f_Oscillospiraceae;g_Colidextribacter | k_Bacteria;p_Bacteroidota;c_Bacteroidia;o_Bacteroidales;f_Marinifilaceae;g_Odoribacter | 0.668569011 | 6.70E-04 | 0.071439879 |
| k_Bacteria;p_Proteobacteria;c_Gammaproteobacteria;o_Burkholderiales;f_Oxalobacteraceae;g_Oxalobacter | k_Bacteria;p_Bacteroidota;c_Bacteroidia;o_Bacteroidales;f_Marinifilaceae;g_Odoribacter | 0.646082296 | 0.001161084 | 0.09580019 |
| k_Bacteria;p_Proteobacteria;c_Gammaproteobacteria;o_Burkholderiales;f_Sutterellaceae;g_Sutterella | k_Bacteria;p_Bacteroidota;c_Bacteroidia;o_Bacteroidales;f_Marinifilaceae;g_Odoribacter | 0.706597807 | 2.37E-04 | 0.043095292 |
| k_Bacteria;p_Firmicutes;c_Bacilli;o_Erysipelotrichales;f_Erysipelotrichaceae;g_Holdemanella | k_Bacteria;p_Bacteroidota;c_Bacteroidia;o_Bacteroidales;f_Prevotellaceae;g_Prevotella | 0.641186308 | 0.001301145 | 0.099098302 |

| | | | | |
|---|---|---|---|---|
| k_Bacteria;p_Firmicutes;c_Clostridia;o_Oscillospirales;f_Oscillospiraceae;g_Colidextribacter | k_Bacteria;p_Bacteroidota;c_Bacteroidia;o_Bacteroidales;f_Prevotellaceae;g_Prevotella | 0.671812157 | 6.17E-04 | 0.071439879 |
| k_Bacteria;p_Proteobacteria;c_Gammaproteobacteria;o_Burkholderiales;f_Sutterellaceae;g_Sutterella | k_Bacteria;p_Bacteroidota;c_Bacteroidia;o_Bacteroidales;f_Prevotellaceae;g_Prevotella | 0.687429281 | 4.08E-04 | 0.056819329 |
| k_Bacteria;p_Firmicutes;c_Bacilli;o_Erysipelotrichales;f_Erysipelotrichaceae;g_Clostridium_innocuum | k_Bacteria;p_Bacteroidota;c_Bacteroidia;o_Bacteroidales;f_Rikenellaceae;g_Alistipes | -0.669883321 | 6.48E-04 | 0.071439879 |
| k_Bacteria;p_Firmicutes;c_Clostridia;o_Christensenellales;f_Christensenellaceae;g_Christensenellaceae_R_7 | k_Bacteria;p_Bacteroidota;c_Bacteroidia;o_Bacteroidales;f_Rikenellaceae;g_Alistipes | 0.841914359 | 9.01E-07 | 0.001605142 |
| k_Bacteria;p_Firmicutes;c_Clostridia;o_Clostridia_UCG_014;f_Clostridia_UCG_014;g_Clostridia_UCG_014 | k_Bacteria;p_Bacteroidota;c_Bacteroidia;o_Bacteroidales;f_Rikenellaceae;g_Alistipes | 0.646211197 | 0.001157577 | 0.09580019 |
| k_Bacteria;p_Firmicutes;c_Clostridia;o_Lachnospirales;f_Lachnospiraceae;g_Coprococcus | k_Bacteria;p_Bacteroidota;c_Bacteroidia;o_Bacteroidales;f_Rikenellaceae;g_Alistipes | 0.862640617 | 2.42E-07 | 7.18E-04 |
| k_Bacteria;p_Firmicutes;c_Clostridia;o_Lachnospirales;f_Lachnospiraceae;g_Eubacterium_ventriosum | k_Bacteria;p_Bacteroidota;c_Bacteroidia;o_Bacteroidales;f_Rikenellaceae;g_Alistipes | 0.70128004 | 2.77E-04 | 0.046960008 |
| k_Bacteria;p_Firmicutes;c_Clostridia;o_Lachnospirales;f_Lachnospiraceae;g_Lachnoclostridium | k_Bacteria;p_Bacteroidota;c_Bacteroidia;o_Bacteroidales;f_Rikenellaceae;g_Alistipes | -0.650008499 | 0.001058236 | 0.089808982 |
| k_Bacteria;p_Firmicutes;c_Clostridia;o_Lachnospirales;f_Lachnospirace | k_Bacteria;p_Bacteroidota;c_Bacteroidia;o_Bacteroidales;f_Rikenellac | -0.65344938 | 9.75E- | 0.085985 |

| | | | | |
|---|---|---|---|---|
| ae;g_Ruminococcus_gnavus | eae;g_Alistipes | | 04 | 349 |
| k_Bacteria;p_Firmicutes;c_Clostridia;o_Monoglobales;f_Monoglobaceae;g_Monoglobus | k_Bacteria;p_Bacteroidota;c_Bacteroidia;o_Bacteroidales;f_Rikenellaceae;g_Alistipes | 0.680366079 | 4.93E-04 | 0.061922061 |
| k_Bacteria;p_Firmicutes;c_Clostridia;o_Oscillospirales;f_Eubacterium_coprostanoligenes;g_Eubacterium_coprostanoligenes | k_Bacteria;p_Bacteroidota;c_Bacteroidia;o_Bacteroidales;f_Rikenellaceae;g_Alistipes | 0.642576314 | 0.001259994 | 0.097710696 |
| k_Bacteria;p_Firmicutes;c_Clostridia;o_Oscillospirales;f_Oscillospiraceae;g_Flavonifractor | k_Bacteria;p_Bacteroidota;c_Bacteroidia;o_Bacteroidales;f_Rikenellaceae;g_Alistipes | -0.751875284 | 5.46E-05 | 0.024341767 |
| k_Bacteria;p_Firmicutes;c_Clostridia;o_Oscillospirales;f_Oscillospiraceae;g_NK4A214 | k_Bacteria;p_Bacteroidota;c_Bacteroidia;o_Bacteroidales;f_Rikenellaceae;g_Alistipes | 0.743638778 | 7.29E-05 | 0.024755225 |
| k_Bacteria;p_Firmicutes;c_Clostridia;o_Peptococcales;f_Peptococcaceae;g_Peptococcus | k_Bacteria;p_Bacteroidota;c_Bacteroidia;o_Bacteroidales;f_Rikenellaceae;g_Alistipes | 0.651705022 | 0.001016258 | 0.087921125 |
| k_Bacteria;p_Firmicutes;c_Clostridia;o_Peptostreptococcales_Tissierellales;f_Anaerovoracaceae;g_Family_XIII_AD3011 | k_Bacteria;p_Bacteroidota;c_Bacteroidia;o_Bacteroidales;f_Rikenellaceae;g_Alistipes | 0.743554632 | 7.31E-05 | 0.024755225 |
| k_Bacteria;p_Firmicutes;c_Clostridia;o_Peptostreptococcales_Tissierellales;f_Anaerovoracaceae;g_Family_XIII_UCG_001 | k_Bacteria;p_Bacteroidota;c_Bacteroidia;o_Bacteroidales;f_Rikenellaceae;g_Alistipes | 0.687414886 | 4.08E-04 | 0.056819329 |
| k_Bacteria;p_Proteobacteria;c_Gammaproteobacteria;o_Burkholderiales;f_Sutterellaceae;g_Sutterella | k_Bacteria;p_Bacteroidota;c_Bacteroidia;o_Bacteroidales;f_Rikenellaceae;g_Alistipes | 0.728636476 | 1.20E-04 | 0.0294995 |

| | | | | |
|---|---|---|---|---|
| k_Bacteria;p_Firmicutes;c_Clostridia;o_Oscillospirales;f_Oscillospiraceae;g_NK4A214 | k_Bacteria;p_Firmicutes;c_Bacilli;o_Erysipelotrichales;f_Erysipelatoclostridiaceae;g_Erysipelatoclostridium | -0.711035366 | 2.08E-04 | 0.040239608 |
| k_Bacteria;p_Firmicutes;c_Clostridia;o_Lachnospirales;f_Lachnospiraceae;g_Lachnospiraceae_UCG_010 | k_Bacteria;p_Firmicutes;c_Bacilli;o_Erysipelotrichales;f_Erysipelatoclostridiaceae;g_Erysipelotrichaceae_UCG_003 | 0.682390896 | 4.67E-04 | 0.059983267 |
| k_Bacteria;p_Firmicutes;c_Negativicutes;o_Veillonellales_Selenomonadales;f_Veillonellaceae;g_Dialister | k_Bacteria;p_Firmicutes;c_Bacilli;o_Erysipelotrichales;f_Erysipelatoclostridiaceae;g_Erysipelotrichaceae_UCG_003 | 0.643380303 | 0.001236702 | 0.097710696 |
| k_Bacteria;p_Firmicutes;c_Clostridia;o_Christensenellales;f_Christensenellaceae;g_Christensenellaceae_R_7 | k_Bacteria;p_Firmicutes;c_Bacilli;o_Erysipelotrichales;f_Erysipelotrichaceae;g_Clostridium_innocuum | -0.693106912 | 3.49E-04 | 0.051798987 |
| k_Bacteria;p_Firmicutes;c_Clostridia;o_Lachnospirales;f_Lachnospiraceae;g_Lachnospiraceae_ND3007 | k_Bacteria;p_Firmicutes;c_Bacilli;o_Erysipelotrichales;f_Erysipelotrichaceae;g_Clostridium_innocuum | -0.656643594 | 9.02E-04 | 0.081651541 |
| k_Bacteria;p_Firmicutes;c_Clostridia;o_Lachnospirales;f_Lachnospiraceae;g_Ruminococcus_gnavus | k_Bacteria;p_Firmicutes;c_Bacilli;o_Erysipelotrichales;f_Erysipelotrichaceae;g_Clostridium_innocuum | 0.669599817 | 6.53E-04 | 0.071439879 |
| k_Bacteria;p_Firmicutes;c_Clostridia;o_Oscillospirales;f_Ruminococcaceae;g_Ruminococcus | k_Bacteria;p_Firmicutes;c_Bacilli;o_Erysipelotrichales;f_Erysipelotrichaceae;g_Clostridium_innocuum | -0.670357881 | 6.41E-04 | 0.071439879 |
| k_Bacteria;p_Firmicutes;c_Clostridia;o_Oscillospirales;f_Ruminococcaceae;g_Subdoligranulum | k_Bacteria;p_Firmicutes;c_Bacilli;o_Erysipelotrichales;f_Erysipelotrichaceae;g_Clostridium_innocuum | -0.728055055 | 1.22E-04 | 0.0294995 |
| k_Bacteria;p_Firmicutes;c_Clostridia;o_Clostridia_UCG_014;f_Clostrid | k_Bacteria;p_Firmicutes;c_Bacilli;o_Erysipelotrichales;f_Erysipelotric | 0.67464905 | 5.73E- | 0.069045 |

| | | | | |
|---|---|---|---|---|
| ia_UCG_014;g_Clostridia_UCG_014 | haceae;g_Holdemanella | | 04 | 501 |
| k_Bacteria;p_Firmicutes;c_Clostridia;o_Peptococcales;f_Peptococcaceae;g_Peptococcus | k_Bacteria;p_Firmicutes;c_Bacilli;o_Erysipelotrichales;f_Erysipelotrichaceae;g_Holdemanella | 0.796862097 | 9.06E-06 | 0.010087094 |
| k_Bacteria;p_Proteobacteria;c_Gammaproteobacteria;o_Pasteurellales;f_Pasteurellaceae;g_Haemophilus | k_Bacteria;p_Firmicutes;c_Bacilli;o_Erysipelotrichales;f_Erysipelotrichaceae;g_Turicibacter | 0.693552273 | 3.44E-04 | 0.051798987 |
| k_Bacteria;p_Firmicutes;c_Clostridia;o_Lachnospirales;f_Lachnospiraceae;g_Ruminococcus_gnavus | k_Bacteria;p_Firmicutes;c_Bacilli;o_Lactobacillales;f_Streptococcaceae;g_Streptococcus | 0.66839233 | 6.73E-04 | 0.071439879 |
| k_Bacteria;p_Firmicutes;c_Clostridia;o_Clostridia_UCG_014;f_Clostridia_UCG_014;g_Clostridia_UCG_014 | k_Bacteria;p_Firmicutes;c_Clostridia;o_Christensenellales;f_Christensenellaceae;g_Christensenellaceae_R_7 | 0.684940867 | 4.36E-04 | 0.058927072 |
| k_Bacteria;p_Firmicutes;c_Clostridia;o_Lachnospirales;f_Lachnospiraceae;g_Coprococcus | k_Bacteria;p_Firmicutes;c_Clostridia;o_Christensenellales;f_Christensenellaceae;g_Christensenellaceae_R_7 | 0.753217698 | 5.21E-05 | 0.024341767 |
| k_Bacteria;p_Firmicutes;c_Clostridia;o_Lachnospirales;f_Lachnospiraceae;g_Lachnoclostridium | k_Bacteria;p_Firmicutes;c_Clostridia;o_Christensenellales;f_Christensenellaceae;g_Christensenellaceae_R_7 | -0.665696667 | 7.21E-04 | 0.072182838 |
| k_Bacteria;p_Firmicutes;c_Clostridia;o_Monoglobales;f_Monoglobaceae;g_Monoglobus | k_Bacteria;p_Firmicutes;c_Clostridia;o_Christensenellales;f_Christensenellaceae;g_Christensenellaceae_R_7 | 0.687815032 | 4.04E-04 | 0.056819329 |
| k_Bacteria;p_Firmicutes;c_Clostridia;o_Oscillospirales;f_Oscillospiraceae;g_NK4A214 | k_Bacteria;p_Firmicutes;c_Clostridia;o_Christensenellales;f_Christensenellaceae;g_Christensenellaceae_R_7 | 0.672801322 | 6.02E-04 | 0.071439879 |

| | | | | |
|---|---|---|---|---|
| k_Bacteria;p_Firmicutes;c_Clostridia;o_Peptococcales;f_Peptococcaceae;g_Peptococcus | k_Bacteria;p_Firmicutes;c_Clostridia;o_Christensenellales;f_Christensenellaceae;g_Christensenellaceae_R_7 | 0.736713721 | 9.22E-05 | 0.026512123 |
| k_Bacteria;p_Firmicutes;c_Clostridia;o_Peptostreptococcales_Tissierellales;f_Anaerovoracaceae;g_Family_XIII_AD3011 | k_Bacteria;p_Firmicutes;c_Clostridia;o_Christensenellales;f_Christensenellaceae;g_Christensenellaceae_R_7 | 0.760370618 | 4.01E-05 | 0.01984343 |
| k_Bacteria;p_Firmicutes;c_Clostridia;o_Peptostreptococcales_Tissierellales;f_Anaerovoracaceae;g_Family_XIII_UCG_001 | k_Bacteria;p_Firmicutes;c_Clostridia;o_Christensenellales;f_Christensenellaceae;g_Christensenellaceae_R_7 | 0.658354806 | 8.65E-04 | 0.081632595 |
| k_Bacteria;p_Proteobacteria;c_Gammaproteobacteria;o_Burkholderiales;f_Sutterellaceae;g_Sutterella | k_Bacteria;p_Firmicutes;c_Clostridia;o_Christensenellales;f_Christensenellaceae;g_Christensenellaceae_R_7 | 0.711934448 | 2.02E-04 | 0.040038795 |
| k_Bacteria;p_Firmicutes;c_Clostridia;o_Clostridia_vadinBB60;f_Clostridia_vadinBB60;g_Clostridia_vadinBB60 | k_Bacteria;p_Firmicutes;c_Clostridia;o_Clostridia_UCG_014;f_Clostridia_UCG_014;g_Clostridia_UCG_014 | 0.868858754 | 1.56E-07 | 6.96E-04 |
| k_Bacteria;p_Firmicutes;c_Clostridia;o_Lachnospirales;f_Lachnospiraceae;g_Coprococcus | k_Bacteria;p_Firmicutes;c_Clostridia;o_Clostridia_UCG_014;f_Clostridia_UCG_014;g_Clostridia_UCG_014 | 0.664170153 | 7.49E-04 | 0.073078264 |
| k_Bacteria;p_Firmicutes;c_Clostridia;o_Oscillospirales;f_Ruminococcaceae;g_Eubacterium_siraeum | k_Bacteria;p_Firmicutes;c_Clostridia;o_Clostridia_UCG_014;f_Clostridia_UCG_014;g_Clostridia_UCG_014 | 0.821635012 | 2.75E-06 | 0.003505798 |
| k_Bacteria;p_Firmicutes;c_Clostridia;o_Peptococcales;f_Peptococcaceae;g_Peptococcus | k_Bacteria;p_Firmicutes;c_Clostridia;o_Clostridia_UCG_014;f_Clostridia_UCG_014;g_Clostridia_UCG_014 | 0.735755574 | 9.52E-05 | 0.026516695 |
| k_Bacteria;p_Firmicutes;c_Clostridia;o_Peptostreptococcales_Tissierella | k_Bacteria;p_Firmicutes;c_Clostridia;o_Clostridia_UCG_014;f_Clostri | 0.836498761 | 1.23E- | 0.001829 |

| | | | | |
|---|---|---|---|---|
| les;f_Anaerovoracaceae;g_Family_XIII_UCG_001 | dia_UCG_014;g_Clostridia_UCG_014 | | 06 | 36 |
| k_Bacteria;p_Proteobacteria;c_Gammaproteobacteria;o_Burkholderiales;f_Oxalobacteraceae;g_Oxalobacter | k_Bacteria;p_Firmicutes;c_Clostridia;o_Clostridia_UCG_014;f_Clostridia_UCG_014;g_Clostridia_UCG_014 | 0.85171424 | 4.96E-07 | 0.001104342 |
| k_Bacteria;p_Firmicutes;c_Clostridia;o_Lachnospirales;f_Lachnospiraceae;g_Coprococcus | k_Bacteria;p_Firmicutes;c_Clostridia;o_Clostridia_vadinBB60;f_Clostridia_vadinBB60;g_Clostridia_vadinBB60 | 0.705558112 | 2.44E-04 | 0.043542509 |
| k_Bacteria;p_Firmicutes;c_Clostridia;o_Oscillospirales;f_Oscillospiraceae;g_Flavonifractor | k_Bacteria;p_Firmicutes;c_Clostridia;o_Clostridia_vadinBB60;f_Clostridia_vadinBB60;g_Clostridia_vadinBB60 | -0.739975028 | 8.27E-05 | 0.024755225 |
| k_Bacteria;p_Firmicutes;c_Clostridia;o_Oscillospirales;f_Ruminococcaceae;g_Eubacterium_siraeum | k_Bacteria;p_Firmicutes;c_Clostridia;o_Clostridia_vadinBB60;f_Clostridia_vadinBB60;g_Clostridia_vadinBB60 | 0.676103534 | 5.52E-04 | 0.067386434 |
| k_Bacteria;p_Firmicutes;c_Clostridia;o_Peptococcales;f_Peptococcaceae;g_Peptococcus | k_Bacteria;p_Firmicutes;c_Clostridia;o_Clostridia_vadinBB60;f_Clostridia_vadinBB60;g_Clostridia_vadinBB60 | 0.6666118 | 7.04E-04 | 0.071736373 |
| k_Bacteria;p_Firmicutes;c_Clostridia;o_Peptostreptococcales_Tissierellales;f_Anaerovoracaceae;g_Family_XIII_UCG_001 | k_Bacteria;p_Firmicutes;c_Clostridia;o_Clostridia_vadinBB60;f_Clostridia_vadinBB60;g_Clostridia_vadinBB60 | 0.722832072 | 1.45E-04 | 0.033357 |
| k_Bacteria;p_Proteobacteria;c_Gammaproteobacteria;o_Burkholderiales;f_Oxalobacteraceae;g_Oxalobacter | k_Bacteria;p_Firmicutes;c_Clostridia;o_Clostridia_vadinBB60;f_Clostridia_vadinBB60;g_Clostridia_vadinBB60 | 0.697200257 | 3.11E-04 | 0.049464931 |
| k_Bacteria;p_Firmicutes;c_Clostridia;o_Lachnospirales;f_Lachnospiraceae;g_Lachnospira | k_Bacteria;p_Firmicutes;c_Clostridia;o_Lachnospirales;f_Lachnospiraceae;g_Agathobacter | 0.668876081 | 6.65E-04 | 0.071439879 |

| | | | | |
|---|---|---|---|---|
| k_Bacteria;p_Proteobacteria;c_Gammaproteobacteria;o_Enterobacterales ;f_Enterobacteriaceae;g_Escherichia_Shigella | k_Bacteria;p_Firmicutes;c_Clostridia;o_Lachnospirales;f_Lachnospiraceae;g_Anaerostipes | -0.663883239 | 7.54E-04 | 0.073078264 |
| k_Bacteria;p_Proteobacteria;c_Gammaproteobacteria;o_Pseudomonadales;f_Moraxellaceae;g_Acinetobacter | k_Bacteria;p_Firmicutes;c_Clostridia;o_Lachnospirales;f_Lachnospiraceae;g_Blautia | -0.656999891 | 8.94E-04 | 0.081651541 |
| k_Bacteria;p_Firmicutes;c_Clostridia;o_Oscillospirales;f_Oscillospiraceae;g_Flavonifractor | k_Bacteria;p_Firmicutes;c_Clostridia;o_Lachnospirales;f_Lachnospiraceae;g_Coprococcus | -0.731777713 | 1.09E-04 | 0.029315343 |
| k_Bacteria;p_Firmicutes;c_Clostridia;o_Peptostreptococcales_Tissierellales;f_Anaerovoracaceae;g_Family_XIII_AD3011 | k_Bacteria;p_Firmicutes;c_Clostridia;o_Lachnospirales;f_Lachnospiraceae;g_Coprococcus | 0.699114424 | 2.94E-04 | 0.047845589 |
| k_Bacteria;p_Proteobacteria;c_Gammaproteobacteria;o_Burkholderiales;f_Sutterellaceae;g_Sutterella | k_Bacteria;p_Firmicutes;c_Clostridia;o_Lachnospirales;f_Lachnospiraceae;g_Coprococcus | 0.719677356 | 1.60E-04 | 0.035555507 |
| k_Bacteria;p_Firmicutes;c_Clostridia;o_Oscillospirales;f_Ruminococcaceae;g_Candidatus_Soleaferrea | k_Bacteria;p_Firmicutes;c_Clostridia;o_Lachnospirales;f_Lachnospiraceae;g_Dorea | 0.669261652 | 6.59E-04 | 0.071439879 |
| k_Bacteria;p_Firmicutes;c_Clostridia;o_Lachnospirales;f_Lachnospiraceae;g_Lachnospiraceae_FCS020 | k_Bacteria;p_Firmicutes;c_Clostridia;o_Lachnospirales;f_Lachnospiraceae;g_Eubacterium_ventriosum | 0.656466741 | 9.06E-04 | 0.081651541 |
| k_Bacteria;p_Firmicutes;c_Clostridia;o_Monoglobales;f_Monoglobaceae;g_Monoglobus | k_Bacteria;p_Firmicutes;c_Clostridia;o_Lachnospirales;f_Lachnospiraceae;g_Eubacterium_ventriosum | 0.66878803 | 6.67E-04 | 0.071439879 |
| k_Bacteria;p_Firmicutes;c_Clostridia;o_Lachnospirales;f_Lachnospirace | k_Bacteria;p_Firmicutes;c_Clostridia;o_Lachnospirales;f_Lachnospira | 0.700948429 | 2.79E- | 0.046960 |

| | | | | |
|---|---|---|---|---|
| ae;g_Lachnospiraceae_ND3007 | ceae;g_Fusicatenibacter | | 04 | 008 |
| k_Bacteria;p_Firmicutes;c_Clostridia;o_Monoglobales;f_Monoglobaceae;g_Monoglobus | k_Bacteria;p_Firmicutes;c_Clostridia;o_Lachnospirales;f_Lachnospiraceae;g_Fusicatenibacter | 0.645419536 | 0.001179258 | 0.096037059 |
| k_Bacteria;p_Firmicutes;c_Clostridia;o_Oscillospirales;f_Ruminococcaceae;g_Ruminococcus | k_Bacteria;p_Firmicutes;c_Clostridia;o_Lachnospirales;f_Lachnospiraceae;g_Fusicatenibacter | 0.739729546 | 8.33E-05 | 0.024755225 |
| k_Bacteria;p_Firmicutes;c_Clostridia;o_Lachnospirales;f_Lachnospiraceae;g_Lachnospiraceae_FCS020 | k_Bacteria;p_Firmicutes;c_Clostridia;o_Lachnospirales;f_Lachnospiraceae;g_Lachnoclostridium | -0.664503022 | 7.43E-04 | 0.073078264 |
| k_Bacteria;p_Firmicutes;c_Clostridia;o_Monoglobales;f_Monoglobaceae;g_Monoglobus | k_Bacteria;p_Firmicutes;c_Clostridia;o_Lachnospirales;f_Lachnospiraceae;g_Lachnospiraceae_ND3007 | 0.777646047 | 2.05E-05 | 0.015132952 |
| k_Bacteria;p_Firmicutes;c_Clostridia;o_Oscillospirales;f_Ruminococcaceae;g_Ruminococcus | k_Bacteria;p_Firmicutes;c_Clostridia;o_Lachnospirales;f_Lachnospiraceae;g_Lachnospiraceae_ND3007 | 0.744951166 | 6.97E-05 | 0.024755225 |
| k_Bacteria;p_Firmicutes;c_Clostridia;o_Oscillospirales;f_Eubacterium_coprostanoligenes;g_Eubacterium_coprostanoligenes | k_Bacteria;p_Firmicutes;c_Clostridia;o_Lachnospirales;f_Lachnospiraceae;g_Lachnospiraceae_UCG_004 | 0.658111421 | 8.70E-04 | 0.081632595 |
| k_Bacteria;p_Firmicutes;c_Clostridia;o_Oscillospirales;f_Oscillospiraceae;g_Colidextribacter | k_Bacteria;p_Firmicutes;c_Clostridia;o_Lachnospirales;f_Lachnospiraceae;g_Lachnospiraceae_UCG_010 | 0.775819311 | 2.21E-05 | 0.015132952 |
| k_Bacteria;p_Firmicutes;c_Clostridia;o_Oscillospirales;f_Ruminococcaceae;g_Subdoligranulum | k_Bacteria;p_Firmicutes;c_Clostridia;o_Lachnospirales;f_Lachnospiraceae;g_Lachnospiraceae_UCG_010 | 0.64341966 | 0.001235571 | 0.097710696 |

| | | | | |
|---|---|---|---|---|
| k_Bacteria;p_Firmicutes;c_Clostridia;o_Lachnospirales;f_Lachnospiraceae;g_Ruminococcus_torques | k_Bacteria;p_Firmicutes;c_Clostridia;o_Lachnospirales;f_Lachnospiraceae;g_Roseburia | 0.766345357 | 3.20E-05 | 0.016991198 |
| k_Bacteria;p_Firmicutes;c_Clostridia;o_Oscillospirales;f_Ruminococcaceae;g_Subdoligranulum | k_Bacteria;p_Firmicutes;c_Clostridia;o_Lachnospirales;f_Lachnospiraceae;g_Ruminococcus_gnavus | -0.650820288 | 0.001037969 | 0.088935977 |
| k_Bacteria;p_Firmicutes;c_Negativicutes;o_Veillonellales_Selenomonadales;f_Veillonellaceae;g_Dialister | k_Bacteria;p_Firmicutes;c_Clostridia;o_Lachnospirales;f_Lachnospiraceae;g_Ruminococcus_gnavus | -0.683645952 | 4.52E-04 | 0.059983267 |
| k_Bacteria;p_Firmicutes;c_Clostridia;o_Oscillospirales;f_Oscillospiraceae;g_NK4A214 | k_Bacteria;p_Firmicutes;c_Clostridia;o_Monoglobales;f_Monoglobaceae;g_Monoglobus | 0.696083697 | 3.21E-04 | 0.050156263 |
| k_Bacteria;p_Firmicutes;c_Clostridia;o_Oscillospirales;f_Ruminococcaceae;g_Ruminococcus | k_Bacteria;p_Firmicutes;c_Clostridia;o_Monoglobales;f_Monoglobaceae;g_Monoglobus | 0.714993749 | 1.84E-04 | 0.038196885 |
| k_Bacteria;p_Firmicutes;c_Clostridia;o_Peptostreptococcales_Tissierellales;f_Peptostreptococcaceae;g_Romboutsia | k_Bacteria;p_Firmicutes;c_Clostridia;o_Monoglobales;f_Monoglobaceae;g_Monoglobus | 0.71227169 | 2.00E-04 | 0.040038795 |
| k_Bacteria;p_Firmicutes;c_Clostridia;o_Oscillospirales;f_Oscillospiraceae;g_NK4A214 | k_Bacteria;p_Firmicutes;c_Clostridia;o_Oscillospirales;f_Eubacterium_coprostanoligenes;g_Eubacterium_coprostanoligenes | 0.728790999 | 1.20E-04 | 0.0294995 |
| k_Bacteria;p_Proteobacteria;c_Gammaproteobacteria;o_Burkholderiales;f_Sutterellaceae;g_Sutterella | k_Bacteria;p_Firmicutes;c_Clostridia;o_Oscillospirales;f_Eubacterium_coprostanoligenes;g_Eubacterium_coprostanoligenes | 0.739875047 | 8.29E-05 | 0.024755225 |
| k_Bacteria;p_Firmicutes;c_Negativicutes;o_Veillonellales_Selenomonad | k_Bacteria;p_Firmicutes;c_Clostridia;o_Oscillospirales;f_Oscillospirac | 0.783583413 | 1.61E- | 0.013720 |

| | | | | |
|---|---|---|---|---|
| ales;f_Veillonellaceae;g_Dialister | eae;g_Colidextribacter | | 05 | 782 |
| k_Bacteria;p_Firmicutes;c_Clostridia;o_Peptostreptococcales_Tissierellales;f_Anaerovoracaceae;g_Family_XIII_UCG_001 | k_Bacteria;p_Firmicutes;c_Clostridia;o_Oscillospirales;f_Oscillospiraceae;g_Flavonifractor | -0.708141479 | 2.26E-04 | 0.042928084 |
| k_Bacteria;p_Proteobacteria;c_Gammaproteobacteria;o_Burkholderiales;f_Sutterellaceae;g_Sutterella | k_Bacteria;p_Firmicutes;c_Clostridia;o_Oscillospirales;f_Oscillospiraceae;g_Flavonifractor | -0.722532116 | 1.46E-04 | 0.033357 |
| k_Bacteria;p_Firmicutes;c_Clostridia;o_Peptostreptococcales_Tissierellales;f_Anaerovoracaceae;g_Family_XIII_AD3011 | k_Bacteria;p_Firmicutes;c_Clostridia;o_Oscillospirales;f_Oscillospiraceae;g_NK4A214 | 0.656412181 | 9.07E-04 | 0.081651541 |
| k_Bacteria;p_Proteobacteria;c_Gammaproteobacteria;o_Burkholderiales;f_Sutterellaceae;g_Sutterella | k_Bacteria;p_Firmicutes;c_Clostridia;o_Oscillospirales;f_Oscillospiraceae;g_NK4A214 | 0.686183875 | 4.22E-04 | 0.057851922 |
| k_Bacteria;p_Firmicutes;c_Clostridia;o_Peptococcales;f_Peptococcaceae;g_Peptococcus | k_Bacteria;p_Firmicutes;c_Clostridia;o_Oscillospirales;f_Ruminococcaceae;g_Eubacterium_siraeum | 0.666391412 | 7.08E-04 | 0.071736373 |
| k_Bacteria;p_Proteobacteria;c_Gammaproteobacteria;o_Burkholderiales;f_Oxalobacteraceae;g_Oxalobacter | k_Bacteria;p_Firmicutes;c_Clostridia;o_Oscillospirales;f_Ruminococcaceae;g_Eubacterium_siraeum | 0.782321422 | 1.69E-05 | 0.013720782 |
| k_Bacteria;p_Firmicutes;c_Clostridia;o_Peptococcales;f_Peptococcaceae;g_Peptococcus | k_Bacteria;p_Firmicutes;c_Clostridia;o_Oscillospirales;f_Ruminococcaceae;g_Negativibacillus | 0.707377827 | 2.32E-04 | 0.042993409 |
| k_Bacteria;p_Firmicutes;c_Negativicutes;o_Veillonellales_Selenomonadales;f_Veillonellaceae;g_Dialister | k_Bacteria;p_Firmicutes;c_Clostridia;o_Oscillospirales;f_Ruminococcaceae;g_Subdoligranulum | 0.683085253 | 4.59E-04 | 0.059983267 |

| | | | | |
|---|---|---|---|---|
| k_Bacteria;p_Firmicutes;c_Clostridia;o_Peptostreptococcales_Tissierellales;f_Anaerovoracaceae;g_Family_XIII_UCG_001 | k_Bacteria;p_Firmicutes;c_Clostridia;o_Peptococcales;f_Peptococcaceae;g_Peptococcus | 0.676300578 | 5.49E-04 | 0.067386434 |

12
